# Evaluating the impact of remote care in sexual and reproductive health services on outcomes and health inequalities in the UK: a retrospective observational study of routine service-level data

**DOI:** 10.1136/bmjph-2025-004787

**Published:** 2026-09-08

**Authors:** Oliver Stirrup, Nuria Gallego Marquez, Anna Tostevin, Jo Josh, Helen Munro, Melvina Woode Owusu, Danielle Solomon, Iestyn Williams, Andrew Copas, Fiona Burns, Jonathan DC Ross, Louise J Jackson, Jo Gibbs

**Affiliations:** 1 Institute for Global Health, University College London, London, UK; 2 PPIE Lead, London, UK; 3 Hywel Dda University Health Board, Carmarthen, UK; 4 Health Services Management Centre, School of Social Policy and Society, University of Birmingham, Birmingham, UK; 5 Birmingham City University, Birmingham, UK; 6 University of Birmingham, Birmingham, UK; 7 Institute of Applied Health Research, University of Birmingham, Birmingham, UK

**Keywords:** Digital Health, Sexual Health, Epidemiology, Public Health

## Abstract

**Introduction:**

Remote clinical consultations and online postal self-sampling are increasingly established components of sexual and reproductive healthcare provision in the UK. However, there are concerns that uptake and outcomes of remote healthcare may differ by demographic groups and so increase health inequalities.

**Methods:**

We conducted a retrospective analysis of service-level data within two case study areas (CSAs) in England and one in Wales. Primary outcomes were treatment within 6 weeks among chlamydia cases and uptake of long-acting reversible contraception (LARC) among women attending for contraceptive care. Outcomes were compared between 2019 and 2022, during which remote care use increased in all CSAs. We evaluated overall change within each CSA, and whether changes differed by age, gender and sexual behaviour, ethnicity and Index of Multiple Deprivation (IMD).

**Results:**

Analyses included 446 819 individuals across 2019–2022. Comparing 2019 to 2022, there was a small reduction in chlamydia treatment success in CSA-A (relative risk (RR) 0.95, 95% CI 0.93 to 0.98) and small increases in CSA-B (1.12, 1.10 to 1.14) and CSA-C (1.07, 1.03 to 1.12). Changes did not significantly vary by ethnicity or IMD, but treatment success dropped for men who have sex exclusively with women in CSA-A (RR 0.88 vs 0.98 in women) and increased for 16–34 year-olds in CSA-B (p=0.001 across groups). LARC uptake decreased in CSA-A (RR 0.58, 95% CI 0.55 to 0.60) but increased in CSA-B (1.18, 1.15 to 1.21) and CSA-C (1.06, 1.01 to 1.11) as proportion of contraceptive care episodes, although total LARC fittings fell in all CSAs; reduction was greater in women aged 35–49 in CSA-A (p<0.001) and increase was greater in women 16–34 in CSA-B (p<0.001). There was limited evidence for differences by ethnicity or IMD.

**Conclusions:**

The study areas varied in their implementation of remote care and healthcare outcomes, but we found no strong evidence that changes in provision of remote care were associated with increases in health inequity.

**WHAT IS ALREADY KNOWN ON THIS TOPIC**

**WHAT THIS STUDY ADDS**

- We evaluated health outcomes in three diverse case study areas within the UK and found variation in their implementation of increased remote care, but we did not find strong evidence that these changes were associated with increases in health inequity.

**HOW THIS STUDY MIGHT AFFECT RESEARCH, PRACTICE OR POLICY**

- The uptake and outcomes for remote care in sexual and reproductive healthcare services are primarily determined by local circumstances and implementation, and so the impact of the expansion of such services will be variable across regions.

## Introduction

 Globally, there remains significant unmet need with respect to sexual and reproductive healthcare, resulting in substantial morbidity and mortality.^1^ The WHO recommends improving access to care by decentralising services and complementing in-person services with digital health interventions for sexually transmitted infections (STIs).^2^ Within the UK, over the past 20 years, service provision has adapted to try and meet increasing demand at a time of a reduction in overall budget, including by introducing new models of care.^3^ Online access to testing for chlamydia infection was introduced in the UK as early as 2006^4^ with the aim of increasing access to screening. The proportion of chlamydia testing activity via online postal self-sampling (OPSS) increased substantially in England from 2.6% in 2015 to 38.4% in 2022,^5^ with testing now routinely available for other STIs.^6,7^ This shift in the balance of testing routes reflects policies to address the growing demand for STI testing^8^ while potentially maximising the cost-effectiveness of testing,^9^ given limited resources. In response to increases in demand and financial pressures there was also an increase in the use of telephone consultations in sexual and reproductive health clinics over this period,^10,11^ and the appearance of online-only and remote-only service providers prompted the creation of formal standards to benchmark their safe and effective operation in 2019.^12^ Increases in the provision of remote care were accelerated by the COVID-19 pandemic, with STI testing primarily accessed via remote pathways during the first 4 months of the pandemic in the UK.^13^ Of all sexual health service provision in 2024, 53.2% consultations were delivered face-to-face, 41.6% via the internet (eg, OPSS) and 5.2% via telephone.^8^

The availability of remote services can improve uptake by groups who are less likely to access face-to-face services.^14^ However, there are concerns that the shift to remote provision of STI testing could increase health inequalities^15,16^ and a survey found that most women preferred hybrid or face-to-face only consultations for contraceptive service delivery.^17^ In England most sexual and reproductive health services are commissioned by local authorities, which have been subject to substantial funding cuts in the past decade,^18^ meaning that availability of remote and face-to-face services is not consistent across the country. There has been additional fragmentation of service provision, as HIV services, abortion services, sterilisation/vasectomy and gynaecology are commissioned by NHS England and integrated care boards,^19^ and contraceptive care is also provided by general practitioners.^20^

There is therefore a need to examine different models of service provision and evaluate how the shift in focus to remote care has impacted on access and outcomes, considering the increase in remote clinical consultations by telephone or videocall and including the use of internet-based OPSS services. We conducted a retrospective analysis of the impact of increases in remote care for sexual and reproductive health within two case study areas (CSAs) in England and one in Wales. We focus on the outcomes of timely treatment for chlamydia and uptake of long-acting reversible contraception (LARC) comparing these between 2019, when use of remote care was relatively rare and 2022 when it had become an established component of service delivery in all CSAs.

## Methods

### Study design and data sources

We conducted a retrospective observational study of routinely collected service-level healthcare data from users of clinic-based sexual and reproductive health services and OPSS STI testing in three CSAs (denoted CSA-A/B/C) in England and Wales. The CSAs each comprise a set of local authority districts for which clinic-based sexual and reproductive health services are delivered by a single NHS Trust (table 1). CSA-A and CSA-B cover primarily urban and suburban areas, whereas CSA-C covers a primarily rural area. Anonymised data on consultations, STI testing and treatment, contraceptive care and demographic characteristics were extracted from the routine electronic healthcare records of participating clinic-based and OPSS services. All in-person and remote services (including OPSS) were free of charge for users.

**Table 1:** Summary information regarding each of the case study areas (CSAs)

Characteristics	CSA-A	CSA-B	CSA-C
Region			
Population size for region, age ≥16 years (Census 2021)	1 064 140	886 930	319 414
Local authority districts	2	4	3
Region type	Urban/suburban	Urban	Mostly rural
Clinic services			
Clinic service locations	7 clinic locations	4 clinic locations	7 clinic locations
OPSS services			
Date OPSS first implemented	August 2015	February 2018	Pilot service November 2018, full introduction May 2020
OPSS model and commissioning	Integrated service; local	External provider; wider consortium	National public health programme
Collection of demographic data by OPSS services	Online questionnaire completed by user at test request	Online questionnaire completed by user at test request	Online questionnaire completed by user at test request
OPSS usage restrictions	None	4 kits/year	None
Symptomatic OPSS users	Signposted to clinic but able to access OPSS	Minimally symptomatic able to access OPSS from 2020	Signposted to clinic but able to access OPSS
Online chlamydia treatment pathway available	x	✓	x

OPSS, online postal self-sampling.

We defined ‘remote clinical consultations’ as those conducted by clinic-based service providers via telephone or videocall, with the broader category of ‘remote care’ including online (OPSS), telephone and video clinical consultations. The main statistical analyses compare outcomes between 2019 and 2022. Data on primary outcomes and chlamydia positivity are plotted for 2019–2022 to provide additional context. We also directly compared outcomes between those who received initial face-to-face or remote contact within 2022. Analyses included all individuals aged ≥16 years who accessed participating services.

For CSA-A OPSS was provided by and fully integrated with the local sexual health service and had been running since 2015, whereas for CSA-B and CSA-C OPSS was run by separate organisations (a commercial provider in CSA-B and a national public health organisation in the case of CSA-C). In CSA-B the OPSS service started in 2018. OPSS testing comprised a pilot service in CSA-C in 2019, which was formally introduced in May 2020. All OPSS services required users to complete an online questionnaire to receive a test kit. For CSA-B and CSA-C people with positive test results on OPSS were transferred to local clinics for further care, although postal treatment was available for uncomplicated chlamydia cases in CSA-B. Testing activity was considered to have taken place when an OPSS sample was returned. Clinic datasets include all service users, but OPSS datasets for CSA-B and CSA-C were limited to individuals residing in the core catchment areas (based on local authorities) of the clinic-based service. There was not person-level linkage between OPSS and clinic datasets for CSA-B and CSA-C, although those presenting to clinics for treatment following a positive OPSS test could be identified in CSA-C (and these records were used to evaluate treatment outcomes in this group).

The datasets for CSA-A and CSA-B overlap with those used in the ASSIST study (Assessing the impact of online self-sampling for STIs & HIV),^21,22^ which included a retrospective analysis of routine healthcare records focusing on the impact of OPSS on STI testing and incidence rates; CSA-A is similar to CSA1 in the previous study but without geographic restriction of home addresses of service users in the present study, CSA-B in the present study represents around half of the area included in CSA2 in the previous study (again without restriction on home addresses) and CSA-C was not included in the ASSIST study. A Strengthening the Reporting of Observational Studies in Epidemiology (STROBE) checklist is provided as online supplemental material.

### Outcomes

Primary outcomes are proportion of chlamydia cases treated within 6 weeks of diagnosis and uptake of LARC. The latter is an indicator of access to a range of highly effective contraceptive methods associated with lower rates of unintended pregnancy.^20,23^ Secondary outcomes are rate of access for contraceptive health services, proportion of gonorrhoea cases treated, time-to-treatment for chlamydia and for gonorrhoea, identification of people attending because of recent sexual assault and identification of 16–17 year-olds with any flags for potential child sexual exploitation (CSE).

For chlamydia and gonorrhoea, diagnoses were based on clinical coding of a new diagnosis within health records. Successful treatment for chlamydia and gonorrhoea was defined as appropriate antibiotic prescription within 1 week prior to or up to 6 weeks after appointment date linked to first positive result (sample return date was used for OPSS cases in CSA-A and CSA-B). Treatment success within 3 weeks of chlamydia diagnosis is also reported as a secondary outcome. Postal treatment for chlamydia was offered for some cases diagnosed on OPSS in CSA-B, with prescription records available for the evaluation of treatment outcomes. For OPSS cases in CSA-B transfer of care to clinic service was also regarded as successful treatment outcome (whether self-reported by patient and/or confirmed by relevant clinic). In CSA-C chlamydia cases on OPSS were referred to clinics for treatment, and their treatment outcomes could be identified in clinic records. Clinical notes relating to treatment provision were used to supplement prescription data for CSA-A. Time-to-treatment for chlamydia and gonorrhoea was evaluated for all cases in CSA-A and CSA-C. In CSA-B, time-to-treatment was evaluated for all clinic cases and for those chlamydia cases who obtained postal treatment following OPSS.

LARC uptake is defined as a ‘new’ or ‘change’ in use of any intrauterine device, intrauterine system, contraceptive injections or implant coded within clinic records and/or a relevant prescription record for any of these within each episode of care. This was evaluated as the proportion of episodes of care involving contraceptive care among cis-gender women or trans men aged 16–49 years. Attendance records were coded as relating to contraceptive service activity based on the presence of any record of change in contraceptive method or status, any prescription of a contraceptive pill or device, or any clinical code relating to contraceptive use or implant removal or contraceptive or family planning advice. Rate of service access linked to contraceptive care was evaluated per 100 person-years (100 PY) for the population of women aged 16–49 years within core catchment areas for each clinic service (details in online supplemental appendix).

The identification of people presenting with recent sexual assault was based on clinical coding for acute sexual assault, or a recorded appointment reason including ‘sexual assault’. Also used were the following questionnaire records: ‘Are you attending today due to rape or sexual assault?’ in CSA-A and ‘Have you recently been the victim of a sexual assault’ for OPSS users in CSA-B. The identification of vulnerable individuals aged 16–17 was based on clinical codes for CSE, domestic violence and sexual assault and a local safeguarding flag in CSA-B. In addition, we used questionnaire data from each site that indicated currently or ever being in a controlling or abusive relationship, any kind of safeguarding referral or recorded involvement with social services (the latter recorded on OPSS for CSA-B).

The outcome of ‘Rates of partner notification following an STI diagnosis’ was included in the study protocol^24^ but has been dropped because of a lack of usable data across the study sites. The planned outcome of ‘repeat termination of pregnancy’ will require use of a different set of data sources, and will be described in a separate publication.

### Episodes of care

For STI care outcomes, analyses were based on episodes of care with duration of 6 weeks (following standard practice for UK Health Security Agency).^25^ Episodes of care started at initial appointment date for clinic datasets and initial online triage date for OPSS test records. This provided a framework for identifying the initial consultation method within any given episode of care. For outcomes relating to contraceptive care, analyses were based on episodes of care with duration of 12 weeks to allow a longer timeframe from initial consultation to LARC uptake. This longer time frame was also used in evaluating identification of people attending for sexual assault or at risk of CSE.

### Demographic variables

Age was categorised by completed years: 16–24, 25–34, 35–49, ≥50 (allowing alignment with national population size data by demographic subgroups). Outcomes were evaluated according to the following groups for most outcomes: all women (including trans women), men who have sex with men (MSM, including trans men and men who have sex with men and with women), and men who have sex exclusively with women (MSEW). We assumed that MSM are likely to be recorded as MSM within clinic databases, while for some sites, information on sexual behaviour/sexual orientation may be left blank for MSEW. Groups were defined based on sexual behaviour where recorded, but we also used recorded sexual orientation if explicit information on sexual behaviour was not available. For the outcomes of LARC uptake and rate of access to contraceptive healthcare, trans men were included in the numerators and denominators but trans women were not. Ethnicity was categorised based on Census categories^26^ as White–British/Irish, White–other, any mixed ethnicity, Asian, Black Caribbean or other, Black African, any other. Index of Multiple Deprivation (IMD) was evaluated using quintile categories.

### Population size of CSAs

Population size was based on National Census 2021 data. Mid-2019 estimates of population size for each lower layer super output area (LSOA) provided by the Office for National Statistics were also used to allow reporting by IMD (this required assumption of a constant sex ratio across LSOAs for each CSA).

### Statistical analysis

For each outcome, we evaluated overall change between 2019 and 2022 within each CSA, and whether changes differed by population characteristics of age, gender and sexual behaviour, ethnicity and IMD. Analyses were conducted separately for each CSA. A set of comparisons was also conducted for those with initial remote (inc. OPSS) versus initial face-to-face contact in 2022. Binary outcomes (eg, treatment success) were analysed using log-link generalised linear models (estimating relative risk (RR)), with cluster-robust standard errors grouped by patient. Time-to-treatment outcomes were analysed using linear regression models. Rate of service access for contraception was analysed using Poisson regression models, with incidence expressed per 100 PY.

For each outcome, overall and within each level of demographic variables, we first compared 2022 to 2019 (or initial remote care vs face-to-face contact) without adjustment for other variables. We then calculated for each demographic variable an interaction P-value testing the null hypothesis that the incidence rate ratio (IRR), RR or mean difference is identical across all levels of the variable. We also conducted multivariable comparisons to investigate the most important factors determining the degree of changes in outcome between time points in each case; for these models it was necessary to use logistic regression models (estimating adjusted ORs) for binary outcomes to ensure computational stability.

Where a variable had <1% missing data in the dataset, affected individuals were excluded from all analyses. There was substantial missing data for ethnicity and IMD, and affected individuals were retained; we used person-level multiple imputation of missing values and combined results with repeat episode of care observations. Ten imputed datasets were created and analysed for each CSA (further details in online supplemental appendix).

The study was designed to have at least 80% power to detect a 10-percentage point difference in each primary outcome between 2019 and 2022 based on preliminary data and assumptions (further details in online supplemental appendix). Data processing and statistical analysis was carried out within the UCL Data Safe Haven and conducted using Stata V.18.0, with graphs generated using ggplot2 for R V.4.3.0.

### Patient and public involvement

The study included a patient and public involvement and engagement (PPIE) representative as a co-applicant, and hence they were involved from the first stages of the research process. A PPIE panel was set up prior to creation of the study protocol and included current users of participating sexual and reproductive health services. The group was chaired by the lead PPIE representative and met regularly. Insights from patients and the public were thus integral to the development of our study design and outcome selection. The PPIE panel gave feedback on data analysis plans for the study. The PPIE group will also help to create lay summary documents and presentations to ensure that the results are presented in a way that is sensitive and accessible to service users.

## Results

The population sizes of the three CSAs were 1.06 million (CSA-A), 0.89 million (CSA-B) and 0.32 million (CSA-C) (table 1). The greatest number of service users per year were observed in CSA-B, although this will have included some double-counting across clinic-attendees and OPSS testers. The proportion of MSM was 2.9% for Census data in CSA-B, substantially higher than CSA-A (1.3%) and CSA-C (1.1%) (online supplemental table 1), and there was corresponding substantial variation in the proportion of service users who were MSM from 4.4% in CSA-C to 17.9% in CSA-B in 2022 (table 2). A full breakdown of available detailed information on sex, gender and sexual behaviour is given in online supplemental table 2. Ethnicity profiles were mixed for both CSA-A and CSA-B but CSA-C was over 90% white British, necessitating use of a simplified set of ethnicity categories for this area. CSA-A showed relatively higher levels of deprivation, with almost half the Census population in the most deprived IMD quintile group.

**Table 2:** Demographic characteristics of sexual and reproductive health service (SRHS) users in each year included in statistical analyses

	CSA-A		CSA-B		CSA-C	
	2019; n (%)	2022; n (%)	2019; n (%)	2022; n (%)	2019; n (%)	2022; n (%)
Overall	74 833	49 934	98 494	92 256	14 294	14 795
Sex grouping						
Women	47 445 (63.4)	30 324 (60.7)	57 953 (58.8)	50 488 (54.7)	11 360 (77.3)	11 636 (78.6)
MSEW	21 471 (28.7)	15 308 (30.7)	24 839 (25.2)	25 284 (27.4)	2915 (19.8)	2503 (16.9)
MSM	5917 (7.9)	4302 (8.6)	15 702 (15.9)	16 484 (17.9)	423 (2.9)	656 (4.4)
Age						
16–24	32 785 (43.8)	18 651 (37.4)	24 438 (24.8)	20 705 (22.4)	6519 (44.4)	5511 (37.2)
25–34	25 293 (33.8)	18 342 (36.7)	45 006 (45.7)	41 052 (44.5)	4226 (28.8)	4789 (32.4)
35–49	13 442 (18.0)	10 350 (20.7)	21 876 (22.2)	22 276 (24.1)	2844 (19.3)	3224 (21.8)
≥50	3313 (4.4)	2591 (5.2)	7174 (7.3)	8223 (8.9)	1109 (7.5)	1271 (8.6)
Ethnicity						
Missing	8907 (11.9)	11 275 (22.6)	27 764 (28.2)	12 732 (13.8)	351 (2.4)	4032 (27.3)
White–British/Irish	33 911 (45.3)	18 985 (38.0)	31 204 (31.7)	33 158 (35.9)	13 492 (91.8)	10 252* (69.3)
White–other	3574 (4.8)	1903 (3.8)	16 364 (16.6)	18 305 (19.8)	582 (4.0)	319 (2.2)
Black African	4383 (5.9)	3117 (6.2)	5420 (5.5)	6154 (6.7)	–	–
Black Carib. or other	7354 (9.8)	5074 (10.2)	4173 (4.2)	4630 (5.0)	–	–
Asian	9543 (12.8)	5019 (10.1)	5070 (5.1)	5720 (6.2)	–	–
Any other	1133 (1.5)	695 (1.4)	2211 (2.2)	4164 (4.5)	273 (1.9)	192 (1.3)
Mixed ethnicity	6028 (8.1)	3866 (7.7)	6288 (6.4)	7393 (8.0)	–	–
IMD						
Missing	5462 (7.3)	3700 (7.4)	882 (0.9)	528 (0.6)	1354 (9.2)	805 (5.4)
First quintile (most deprived)	35 828 (47.9)	23 812 (47.7)	16 918 (17.2)	17 269 (18.7)	1678 (11.4)	1742 (11.8)
Second quintile	14 378 (19.2)	9419 (18.9)	34 976 (35.5)	33 886 (36.7)	2777 (18.9)	2978 (20.1)
Third quintile	10 553 (14.1)	7368 (14.8)	24 105 (24.5)	22 015 (23.9)	4364 (29.7)	4618 (31.2)
Fourth quintile	4444 (5.9)	3088 (6.2)	14 293 (14.5)	12 523 (13.6)	3737 (25.4)	3809 (25.7)
Fifth quintile	4168 (5.6)	2547 (5.1)	7320 (7.4)	6035 (6.5)	788 (5.4)	843 (5.7)

SRHS users included based on any record of clinic attendance or self-sampling testing within each year. For CSA-B and CSA-C, users of both clinic and OPSS services in any given year may be double-counted as we do not have full linkage between datasets.

* Online postal self-sampling records in this year for CSA-C only included a single ‘white’ ethnicity category.

CSA, case study area; IMD, Index of Multiple Deprivation; MSEW, men who have sex exclusively with women; MSM, men who have sex with men; OPSS, online postal self-sampling.

**Figure 1:**
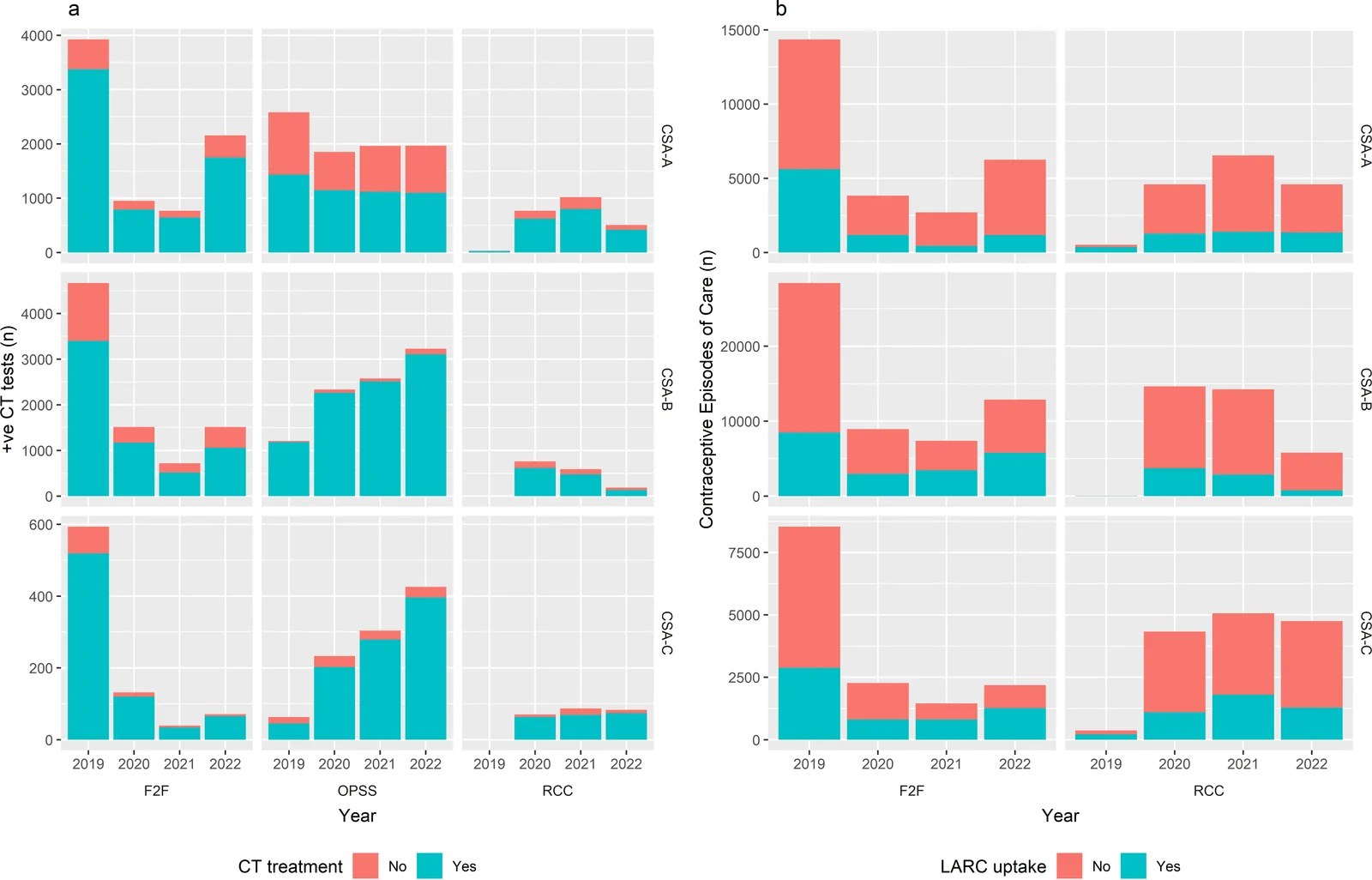
(**a**) Diagnosed cases of chlamydia (CT) and summary of treatment success (within 6 weeks) in 2019–2022. Rows correspond to the three case study areas (CSA-A-C), and data for each outcome are also split according to whether the initial contact in each 6-week episode of care was: face-to-face (F2F), online postal self-sampling (OPSS) or a remote telephone or video clinical consultation (RCC). (**b**) Total counts of episodes of care involving any contraceptive care among women aged 16–49 years in 2019–2022, and proportion of these episodes in which there was any record of new or adjusted long-acting contraception (LARC). Rows correspond to the three CSA-A-C, and data for each outcome are also split according to whether the initial contact in each 12-week episode of care was F2F or RCC.

**Figure 2:**
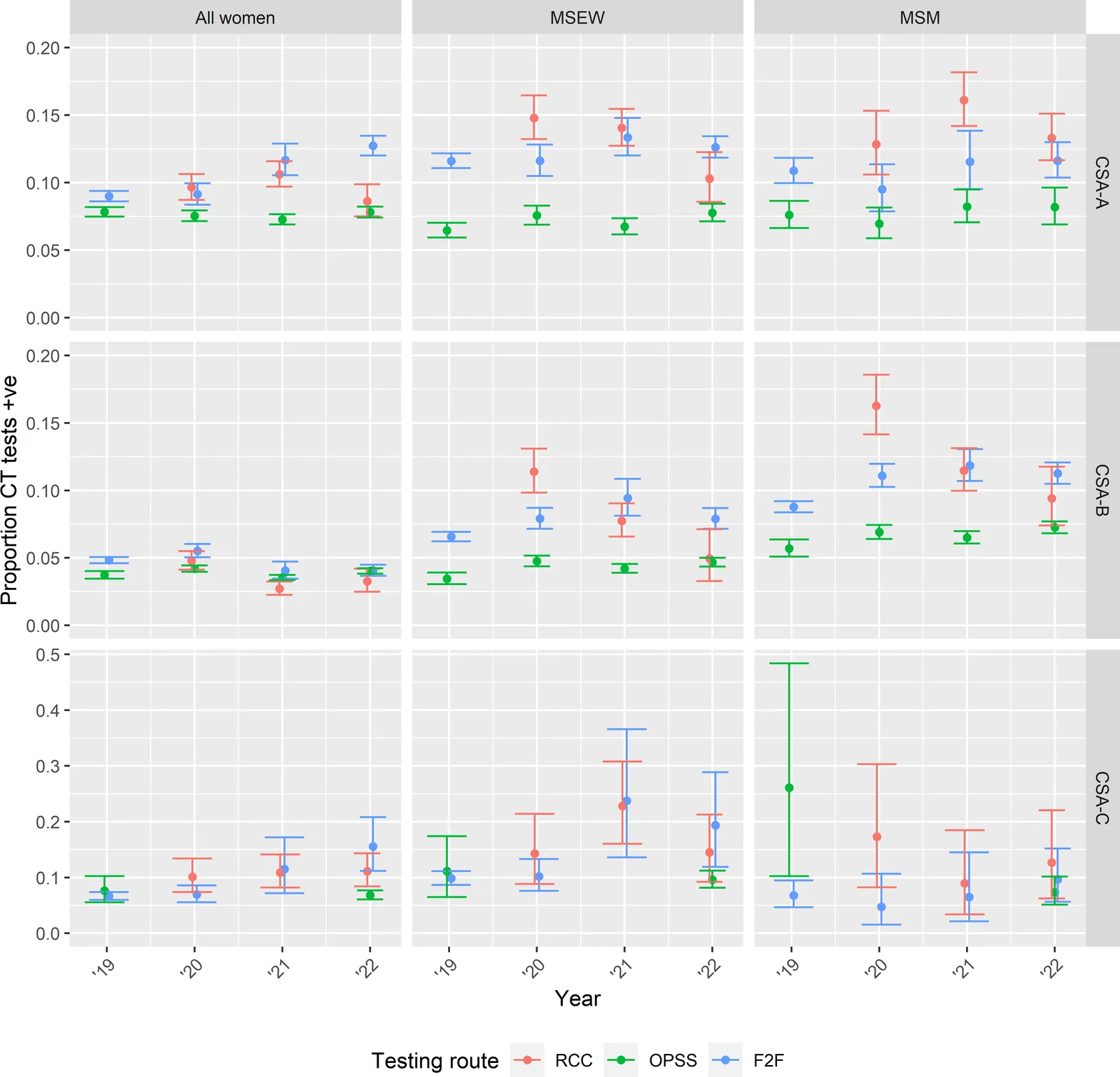
Overall chlamydia (CT) positivity proportion (with 95% CI) within each 6-week episode of care including at least one CT test, faceted by case study area (CSA-A/B/C) and sex and sexual behaviour group: women, men who have sex exclusively with women (MSEW) and men who have sex with men (MSM). Data are also split according to whether the test was online postal self-sampling (OPSS) or clinic-based with initial face-to-face (F2F) or remote clinical consultation (RCC). Positivity is not shown for OPSS testing in CSA-C in 2020–2021, as we do not have a complete record of negative testing activity on OPSS in this period.

OPSS testing for chlamydia was available in all CSAs in 2019, but was still in the process of becoming an established part of service provision in CSA-B and CSA-C where an increase in OPSS usage was seen through to 2022. All areas saw a substantial fall in diagnoses resulting from face-to-face initial contact in 2020 and 2021, with partial recovery in these numbers in 2022 (figure 1a). There were a small number of chlamydia diagnoses following an initial remote telephone or video consultation in CSA-A in 2019, but none in this year in CSA-B and CSA-C. Chlamydia diagnoses following an initial remote telephone or video consultation were observed in all CSAs in 2020–2022, but the numbers were substantially lower than those resulting from OPSS testing in all years considered. Chlamydia positivity was consistently lower for OPSS than for clinic-based testing (figure 2), with the only exceptions observed among the 2019 OPSS pilot phase in CSA-C and among women in CSA-B in 2022. Positivity following initial remote clinical care was significantly higher than that following an initial face-to-face consultation among some subgroups in 2020–2021, perhaps reflecting remote risk-based triage during the COVID-19 pandemic.

Overall from 2019 to 2022, there was a small reduction in chlamydia treatment success in CSA-A (RR 0.95, 95% CI 0.93 to 0.98) and small increases in treatment success in CSA-B (1.12, 1.10 to 1.14) and CSA-C (1.07, 1.03 to 1.12) (table 3, multivariable models in online supplemental table 3). Changes did not significantly vary by ethnicity or IMD in any area, but treatment success dropped particularly for MSEW in CSA-A (RR 0.88 vs 0.98 in women, p<0.001 across groups) and increased particularly for 16–34 year-olds in CSA-B (p=0.001 across groups) and for 25–34 year-olds in CSA-C (although non-significant across groups, p=0.312). Similar findings were observed if using a 3-week cut-off for successful treatment (online supplemental table 4).

**Table 3:** Proportion of new chlamydia diagnoses receiving treatment within 6 weeks of diagnosis, comparing the ‘mainly F2F’ (2019) period with ‘mixed consultation model’ (2022) period

	CSA-A		CSA-B		CSA-C	
	2019; n/N (%)	2022; n/N (%) (RR, 95% CI)	2019; n/N (%)	2022; n/N (%) (RR, 95% CI)	2019; n/N (%)	2022; n/N (%) (RR, 95% CI)
Overall outcome	4833/6540 (73.9)	3263/4634 (70.4) (0.95, 0.93 to 0.98)	4579/5878 (77.9)	4297/4928 (87.2) (1.12, 1.10 to 1.14)	564/657 (85.8)	535/580 (92.2) (1.07, 1.03 to 1.12)
Outcome by group						
F2F initial	3373/3927 (85.9)	1748/2158 (81.0)	3397/4669 (72.8)	1058/1514 (69.9)	519/594 (87.4)	65/71 (91.5)
OPSS	1436/2583 (55.6)	1099/1969 (55.8)	1182/1209 (97.8)	3107/3228 (96.3)	45/63 (71.4)	396/426 (93.0)
RCC	24/30 (80.0)	416/507 (82.1)		132/186 (71.0)		74/83 (89.2)
Sex grouping (missing, eq. p value)	0.1%	0.2%, <0.001	0.1%	0.1%, 0.018	0.0%	0.0%, 0.131
Women	2680/3797 (70.6)	1748/2528 (69.1) (0.98, 0.95 to 1.01)	1887/2390 (79.0)	1766/1948 (90.7) (1.15, 1.12 to 1.18)	309/371 (83.3)	317/343 (92.4) (1.11, 1.05 to 1.17)
MSEW	1616/2052 (78.8)	1035/1494 (69.3) (0.88, 0.84 to 0.92)	1242/1582 (78.5)	1097/1236 (88.8) (1.13, 1.09 to 1.17)	226/249 (90.8)	166/178 (93.3) (1.03, 0.97 to 1.09)
MSM	537/691 (77.7)	480/612 (78.4) (1.01, 0.95 to 1.07)	1450/1906 (76.1)	1434/1744 (82.2) (1.08, 1.05 to 1.12)	29/37 (78.4)	52/59 (88.1) (1.12, 0.93 to 1.36)
Age (missing, eq. p value)	0.0%	0.0%, 0.581	0.0%	0.0%, 0.001	0.0%	0.0%, 0.312
16–24	2946/3997 (73.7)	1894/2737 (69.2) (0.94, 0.91 to 0.97)	1649/2104 (78.4)	1566/1747 (89.6) (1.14, 1.11 to 1.18)	392/448 (87.5)	374/403 (92.8) (1.06, 1.02 to 1.11)
25–34	1353/1844 (73.4)	926/1297 (71.4) (0.97, 0.93 to 1.02)	1822/2326 (78.3)	1733/1947 (89.0) (1.14, 1.11 to 1.17)	131/160 (81.9)	116/123 (94.3) (1.15, 1.06 to 1.26)
35–49	427/563 (75.8)	367/501 (73.3) (0.97, 0.90 to 1.04)	875/1138 (76.9)	797/952 (83.7) (1.09, 1.04 to 1.14)	32/38 (84.2)	35/42 (83.3) (0.99, 0.82 to 1.20)
**≥**50	107/136 (78.7)	76/99 (76.8) (0.98, 0.85 to 1.12)	233/310 (75.2)	201/282 (71.3) (0.95, 0.86 to 1.04)	9/11 (81.8)	10/12 (83.3) (1.02, 0.71 to 1.46)
Ethnicity (missing, eq. p value)	7.1%	15.2%, 0.372	27.8%	8.5%, 0.774	0.8%	22.2%, 0.932
White–British/Irish	66.2	64.1 (0.97, 0.93 to 1.01)	78.4	89.6 (1.14, 1.10 to 1.18)	85.7	92.2 (1.08, 1.03 to 1.12)
White–other	76.6	74.0 (0.97, 0.87 to 1.07)	78.8	85.7 (1.09, 1.04 to 1.14)	88.9	93.8 (1.05, 0.87 to 1.28)
Black African	80.6	76.1 (0.94, 0.88 to 1.01)	77.7	87.8 (1.13, 1.05 to 1.22)	–	–
Black Carib/other	84.8	76.1 (0.90, 0.86 to 0.94)	78.8	87.0 (1.10, 1.04 to 1.18)	–	–
Asian	79.2	73.3 (0.93, 0.86 to 0.99)	74.9	82.7 (1.10, 1.02 to 1.19)	–	–
Any other	78.6	74.9 (0.95, 0.77 to 1.17)	80.5	89.7 (1.12, 1.01 to 1.24)	88.9	92.1 (1.04, 0.83 to 1.30)
Mixed	76.4	74.3 (0.97, 0.91 to 1.04)	75.0	83.8 (1.12, 1.05 to 1.19)	–	–
IMD (missing, eq. p value)	5.9%	8.1%, 0.106	0.8%	0.4%, 0.323	9.0%	9.1%, 0.470
First quintile (most deprived)	76.9	71.6 (0.93, 0.90 to 0.96)	76.1	86.9 (1.14, 1.10 to 1.19)	83.1	95.4 (1.15, 1.04 to 1.26)
Second quintile	71.8	72.3 (1.01, 0.96 to 1.06)	79.4	87.7 (1.11, 1.08 to 1.14)	86.2	89.7 (1.04, 0.95 to 1.14)
Third quintile	69.1	68.3 (0.99, 0.92 to 1.06)	78.1	87.9 (1.13, 1.09 to 1.17)	87.2	92.9 (1.07, 1.00 to 1.14)
Fourth quintile	66.8	64.2 (0.96, 0.85 to 1.09)	79.2	85.9 (1.08, 1.03 to 1.14)	84.7	92.6 (1.09, 1.01 to 1.18)
Fifth quintile	68.4	61.8 (0.90, 0.78 to 1.04)	71.3	83.9 (1.18, 1.08 to 1.28)	90.3	89.0 (0.99, 0.84 to 1.16)

eq. p value, ‘interaction’ p value testing equality of RR for change over time across groups; % and RR values calculated using multiple imputation for ethnicity and IMD values, with other missing data excluded.

Change is estimated within categories (RR; 2022 vs 2019).

CSA, case study area; F2F, face-to-face; IMD, Index of Multiple Deprivation; MSEW, men who have sex exclusively with women; MSM, men who have sex with men; OPSS, online postal self-sampling; RCC, remote telephone or video clinical consultation; RR, relative risk.

Within 2022, chlamydia cases diagnosed following initial remote care had worse treatment success versus cases diagnosed following face-to-face contact in CSA-A (RR 0.76, 95% CI 0.73 to 0.78), better treatment success in CSA-B (1.36, 1.31 to 1.40) and there was little difference in CSA-C (1.01, 0.94 to 1.09). MSM had more favourable outcomes following initial remote care in CSA-A and CSA-B, but there was little evidence of differential impact of testing pathway on treatment outcomes according to age, ethnicity or IMD (online supplemental table 5). A similar pattern of results was observed for overall comparisons of gonorrhoea treatment success (online supplemental tables 6,7). For both chlamydia and gonorrhoea, average time-to-treatment increased from 2019 to 2022 in CSA-A, but decreased in CSA-B and CSA-C (table 4 and online supplemental tables 8,9); however, this evaluation is biased for CSA-C for which the starting time point for positive cases on OPSS is their clinic attendance rather than their sample return date (which we could not link to outcomes).

**Table 4:** Summary comparison of secondary outcomes between 2019 and 2022

	CSA-A		CSA-B		CSA-C	
	2019; n/N (%)	2022; n/N (%) (RR, 95% CI)	2019; n/N (%)	2022; n/N (%) (RR, 95% CI)	2019; n/N (%)	2022; n/N (%) (RR, 95% CI)
*Gonorrhoea treatment*						
Overall	2242/2670 (84.0)	2170/2617 (82.9) (0.99, 0.96 to 1.01)	1680/2104 (79.8)	2102/2426 (86.6) (1.09, 1.06 to 1.11)	80/99 (80.8)	118/134 (88.1) (1.09, 0.97 to 1.22)
By pathway						
F2F initial	1743/1964 (88.7)	1375/1549 (88.8)	1276/1681 (75.9)	665/890 (74.7)	69/82 (84.1)	24/25 (96.0)
OPSS	478/680 (70.3)	478/677 (70.6)	404/423 (95.5)	1393/1479 (94.2)	11/17 (64.7)	70/83 (84.3)
RCC	21/26 (80.8)	317/391 (81.1)		44/57 (77.2)		24/26 (92.3)
*Sexual assault*						
Overall	1791/91 844 (2.0)	1474/61 789 (2.4) (1.22, 1.14 to 1.31)	295/127 358 (0.2)	509/117 406 (0.4) (1.87, 1.62 to 2.16)	6/17685 (0.0)	25/17 714 (0.1) (4.16, 1.71 to 10.14)
By pathway						
F2F initial	1637/57 179 (2.9)	1061/23 873 (4.4)	187/99 332 (0.2)	135/47 516 (0.3)	6/16 637 (0.0)	7/4327 (0.2)
OPSS	97/32 766 (0.3)	126/26 336 (0.5)	108/27 911 (0.4)	363/59 351 (0.6)	0/679 (0.0)	14/5750 (0.2)
RCC	57/1899 (3.0)	287/11 580 (2.5)	0/115 (0.0)	11/10 539 (0.1)	0/369 (0.0)	4/7637 (0.1)
*Risk of CSE*						
Overall	167/3573 (4.7)	119/1404 (8.5) (1.81, 1.42 to 2.32)	36/1196 (3.0)	95/848 (11.2) (3.72, 2.57 to 5.40)	7/971 (0.7)	54/791 (6.8) (9.47, 4.33 to 20.72)
	**2019; IR/100PY**	**2022; IR/100PY (IRR, 95% CI)**	**2019; IR/100PY**	**2022; IR/100PY (IRR, 95% CI)**	**2019; IR/100PY**	**2022; IR/100PY (IRR, 95% CI)**
*Contraceptive care*						
	4.5	3.3 (0.7, 0.7 to 0.7)	9.5	6.2 (0.7, 0.6 to 0.7)	13	9.8 (0.8, 0.8 to 0.8)
By pathway						
F2F initial	4.4	1.9	9.5	4.3	12	3.1
RCC	0.16	1.4	0.02	1.9	0.53	6.7
	**2019; mean±SD**	**2022; mean±SD (d, 95% CI)**	**2019; mean±SD**	**2022; mean±SD (d, 95% CI)**	**2019; mean±SD**	**2022; mean±SD (d, 95% CI)**
*Chlamydia time-to-treatment* *						
Overall	7.7±9.3	11.3±8.7 (3.59, 3.17 to 4.01)	4.4±5.6	3.1±3.4 (−1.25, −1.45 to −1.04)	5.4±8.0	1.0±3.9†
*Gonorrhoea time-to-treatment*						
Overall	5.9±7.9	8.4±9.5 (2.46, 1.91 to 3.01)	3.3±5.6	1.7±4.4 (−1.61, −2.06 to −1.17)	4.6±6.4	2.8±4.9†

* Measured from appointment date linked to first positive result for clinic attendees and OPSS testers in CSA-C, or sample return date for OPSS cases in CSA-A and CSA-B, to date of antibiotic prescription (reported for those where this is known).

† Direct comparison with 2019 would be biased for CSA-C, due to lack of knowledge of sample return date for OPSS testers.

CSA, case study area; CSE, child sexual exploitation; d, average difference; F2F, face-to-face; IR/100PY, incidence rate per 100 person-years; IRR, incidence rate ratio; OPSS, online postal self-sampling; RCC, remote telephone or video clinical consultation; RR, relative risk.

In all areas, there was a substantial fall in total number of women accessing contraceptive care through sexual and reproductive health services from 2019 to 2022, with the greatest relative fall among 16–24 year-olds in all cases (table 4 and online supplemental table 10). All CSAs had low levels of remote clinical consultation for provision of any contraceptive care for women in 2019, but in all CSAs most episodes of care started with remote clinical consultation in 2020–2021 (figure 1b). In CSA-A and CSA-B initial face-to-face contact became more common than initial remote clinical consultation in 2022, but majority provision of remote clinical consultations was maintained in CSA-C. There were falls in overall access to contraceptive care in CSA-A (IRR 0.7, 95% CI 0.7 to 0.7), CSA-B (0.7, 0.6 to 0.7) and CSA-C (0.8, 0.8 to 0.8) from 2019 to 2022 (table 4 and online supplemental table 10). LARC uptake as a proportion of episodes of care decreased in CSA-A (RR 0.58, 95% CI 0.55 to 0.60), but increased in CSA-B (1.18, 1.15 to 1.21) and CSA-C (1.06, 1.01 to 1.11) (table 5, multivariable models in online supplemental table 11). There was a greater reduction in LARC uptake in women aged 35–49 in CSA-A (p<0.001) and a greater increase in women 16–34 among attendees in CSA-B (p<0.001), but limited evidence for differences according to ethnicity or IMD—although the proportional reduction was greatest within the most deprived areas in CSA-A. Within 2022, initial contact by remote clinical consultation was associated with higher uptake of LARC in CSA-A (RR 1.56, 1.45 to 1.67), but lower uptake in CSA-B (0.30, 0.28 to 0.32) and CSA-C (0.47, 0.44 to 0.50) (online supplemental table 12); in CSA-B, this was likely due to the use of an online questionnaire and booking system for LARC not captured by our data extraction, and LARC via injectables and implants was also directly booked for in-person appointments in CSA-C.

**Table 5:** Proportion of women aged 16–49 attending for contraceptive care with uptake of long-acting contraception, comparing the ‘mainly F2F’ (2019) period with ‘mixed consultation model’ (2022) period

	CSA-A		CSA-B		CSA-C	
	2019; n/N (%)	2022; n/N (%) (RR, 95% CI)	2019; n/N (%)	2022; n/N (%) (RR, 95% CI)	2019; n/N (%)	2022; n/N (%) (RR, 95% CI)
Overall outcome	5978/14 883 (40.2)	2509/10 851 (23.1) (0.58, 0.55 to 0.60)	8460/28 478 (29.7)	6529/18 671 (35.0) (1.18, 1.15 to 1.21)	3092/8916 (34.7)	2548/6942 (36.7) (1.06, 1.01 to 1.11)
Outcome by group						
F2F initial	5611/14 363 (39.1)	1169/6255 (18.7)	8457/28 427 (29.7)	5762/12 871 (44.8)	2878/8545 (33.7)	1264/2184 (57.9)
RCC	367/520 (70.6)	1340/4596 (29.2)	3/51 (5.9)	767/5800 (13.2)	214/371 (57.8)	1284/4758 (27.0)
Age (missing, eq. p value)	0.0%	0.0%, <0.001	0.0%	0.0%, <0.001	0.0%	0.0%, 0.290
16–24	2541/7774 (32.7)	878/4512 (19.5) (0.60, 0.55 to 0.64)	2456/9552 (25.7)	1613/5166 (31.2) (1.21, 1.15 to 1.28)	1495/4744 (31.5)	983/2965 (33.2) (1.05, 0.98 to 1.13)
25–34	2051/4615 (44.4)	955/3817 (25.0) (0.56, 0.53 to 0.60)	3941/13 674 (28.8)	3019/8831 (34.2) (1.19, 1.14 to 1.23)	864/2468 (35.0)	844/2269 (37.2) (1.06, 0.98 to 1.15)
35–49	1386/2494 (55.6)	676/2522 (26.8) (0.48, 0.45 to 0.52)	2063/5252 (39.3)	1897/4674 (40.6) (1.03, 0.98 to 1.08)	733/1704 (43.0)	721/1708 (42.2) (0.98, 0.91 to 1.06)
Ethnicity (missing, eq. p value)	13.8%	32.8%, 0.026	33.7%	30.0%, 0.613	2.3%	1.8%, 0.779
White–British/Irish	40.8	25.5 (0.62, 0.59 to 0.67)	32.0	36.8 (1.15, 1.10 to 1.20)	34.7	36.8 (1.06, 1.01 to 1.11)
White–other	39.3	24.1 (0.61, 0.51 to 0.73)	28.8	35.7 (1.24, 1.16 to 1.32)	32.6	31.8 (0.98, 0.76 to 1.25)
Black African	41.0	22.5 (0.55, 0.47 to 0.64)	27.3	31.0 (1.14, 1.01 to 1.27)	–	–
Black Carib/other	36.5	17.1 (0.47, 0.40 to 0.55)	24.2	28.9 (1.19, 1.03 to 1.38)	–	–
Asian	41.7	22.8 (0.55, 0.49 to 0.62)	29.3	35.5 (1.21, 1.10 to 1.33)	–	–
Any other	43.9	26.4 (0.60, 0.45 to 0.80)	23.6	29.8 (1.26, 0.99 to 1.61)	36.6	40.6 (1.11, 0.81 to 1.52)
Mixed	38.4	22.3 (0.58, 0.50 to 0.67)	29.3	34.8 (1.19, 1.09 to 1.30)	–	–
IMD (missing, eq. p value)	10.0%	11.4%, 0.019	0.7%	0.7%, 0.785	8.8%	6.1%, 0.057
First quintile (most deprived)	43.3	24.1 (0.56, 0.53 to 0.59)	29.5	33.5 (1.14, 1.07 to 1.22)	36.4	34.0 (0.93, 0.83 to 1.05)
Second quintile	38.2	21.8 (0.57, 0.52 to 0.63)	28.8	34.2 (1.19, 1.13 to 1.24)	37.7	37.6 (1.00, 0.91 to 1.09)
Third quintile	38.3	21.2 (0.55, 0.49 to 0.63)	29.9	36.0 (1.20, 1.14 to 1.27)	34.0	37.8 (1.11, 1.03 to 1.20)
Fourth quintile	33.9	22.8 (0.67, 0.55 to 0.83)	30.9	36.1 (1.17, 1.09 to 1.25)	32.9	36.0 (1.09, 1.01 to 1.19)
Fifth quintile	28.6	22.1 (0.77, 0.63 to 0.95)	31.2	36.6 (1.18, 1.07 to 1.29)	31.1	37.4 (1.20, 0.98 to 1.48)

eq. p value, ‘interaction’ p value testing equality of RR for change over time across groups; % and RR values calculated using multiple imputation for ethnicity and IMD values, with other missing data excluded.

Change is estimated within categories (RR; 2022 vs 2019).

CSA-A, case study area-A; F2F, face-to-face; IMD, Index of Multiple Deprivation; RCC, remote telephone or video clinical consultation; RR, relative risk.

The proportion of all episodes of care in which it was recorded that recent sexual assault was disclosed increased from 2019 to 2022 in all CSAs: from 2.0% to 2.4% (RR 1.22, 95% CI 1.14 to 1.31) in CSA-A, 0.2% to 0.4% (1.87, 1.62 to 2.16) in CSA-B and 0.03% to 0.1% (4.16, 1.71 to 10.14) in CSA-C (table 4 and online supplemental tables 13,14). The substantial differences between the CSAs in proportions observed are likely due to differences in routine enquiry and referral pathways from emergency services and crisis centres. The proportion of all episodes of care relating to under-18s in whom there was any recorded flag for potential CSE increased from 2019 to 2022 in all CSAs: from 4.7% to 8.5% (RR 1.81, 95% CI 1.42 to 2.32) in CSA-A, 3.0% to 11.2% (3.72, 2.57 to 5.40) in CSA-B and 0.7% to 6.8% (9.47, 4.33 to 20.72) in CSA-C (table 4).

## Discussion

Each region considered in this study showed substantial changes in their delivery of sexual and reproductive health services from 2019 to 2022. In CSA-A, OPSS testing for chlamydia was already established in 2019, but in the other CSAs it progressed from a low base to accounting for a clear majority of new diagnoses by 2022. While there was some increase in the number of chlamydia diagnoses following other types of remote clinical consultation in all regions, diagnoses on OPSS were more common. All regions also showed an increase in use of remote clinical consultations to initiate contraceptive care, and this pathway was maintained into 2022 in CSA-C in particular. However, in all CSAs there was a drop between 2019 and 2020 in the total number of consultations for contraception made through each sexual and reproductive health service and as of 2022 this had not returned to prepandemic levels, with the greatest relative fall among 16–24 year-olds. The pattern of changes in sexual health and contraceptive care outcomes was not consistent across CSAs, indicating that they are more strongly affected by local circumstances and implementation than by expansion of remote clinical consultations per se.

While there is evidence that provision of OPSS for STIs can lead to an overall increase in testing levels^22,27^ there have been concerns that differential uptake across socioeconomic groups could widen health inequalities,^15,16^ particularly if other testing routes become more restricted. A systematic review by Iyamu *et al*^28^ found that in the majority of previous studies women and people who were aged over 20 years, with higher incomes, white, heterosexual, cis-gender, and residents of urbanised areas were more likely to use OPSS testing. However, they^28^ also identified some studies in which those with lower incomes, in less urban areas, males and MSM had higher uptake of OPSS, indicating that outcomes may be sensitive to local context. We observed consistently lower positivity for OPSS in comparison to clinic-based testing, indicating that testing was being accessed by individuals at lower risk of STIs.

There is less information in the literature evaluating treatment outcomes for chlamydia diagnoses following introduction of OPSS and how this may affect health equity. One study in Birmingham, England in 2016 found lower treatment completion among OPSS than clinic users (82% vs 88%),^29^ but more recent studies in London^6^ and Baltimore, USA^30^ have found treatment success proportions of ≥96% among chlamydia diagnoses by OPSS. We found a similarly wide range in treatment success for chlamydia across regions. Within region, we did not find evidence of differential changes in treatment outcomes from 2019 to 2022 or for cases following remote care within 2022 according to ethnicity or IMD. We did find some evidence that the introduction of remote care was associated with changes in treatment outcomes by sex and sexual behaviour groups, but the exact pattern varied across study areas.

Increased uptake of LARC was encouraged by UK government policy in the period 2009–2014 leading to a substantial increase in their use in Primary Care.^20^ However, there was a decline in use across England in 2020 and rates of prescribing have not yet recovered to prepandemic levels.^23^ We observed declines in the overall rate of consultations for contraceptive care but an increase in the proportion of women attending in order to access LARC in two of three study areas, in line with national data for England for this period.^31^ Notably, the relative decline in access of contraceptive care was greatest among 16–24 years in all CSAs, while the rate of access for 35–49 year-olds was either level or showed only a small decrease. All of our study areas offered access to contraceptive services via either initial face-to-face or remote consultation pathways in 2022, and we found limited impact of differential changes in LARC uptake according to ethnicity or IMD. Contraception is more commonly accessed via primary care in the UK,^31^ but those who access care through dedicated sexual and reproductive health clinics are more likely to be at high risk of poor sexual health.^32^

Our study areas all displayed substantial increases in the proportion of service users recorded as attending due to recent sexual assault and the proportion of under-18s at-risk for CSE. We believe that this is the result of more consistent and standardised recording of relevant fields within digital records systems of both clinic-based and OPSS service providers. This suggests that participating providers are increasingly using an information-gathering approach^33^ in identifying vulnerable service users. A survey of UK sexual health services in 2022–2023 found highly variable practice in the identification of sexual and domestic violence among service users.^34^

A limitation of this study is that we are missing data on ethnicity for a substantial number of individuals, which was addressed through multiple imputation in the statistical analyses. The levels of missing data varied significantly between time periods for all CSAs; in CSA-B this can be attributed largely to more comprehensive recording of ethnicity in the online questionnaires used by the OPSS service, while in CSA-C ethnicity was recorded in a lower proportion of OPSS users than among clinic attendees. This could be flagged as a data quality issue for services to address to ensure that they are able to fully evaluate potential disparities in access to care and outcomes by ethnicity. Our analysis did not aim to evaluate differences in barriers to care or health outcomes associated with specific intersectional identities, but the presence of missing data for key variables would make this substantially more difficult to achieve.

Another limitation is the fact that this evaluation is restricted to outcomes for users of sexual and reproductive health services within each area, meaning that trends and outcomes for those seeking care through general practitioners^5,20,23^ could not be assessed. Within CSA-B there was no direct linkage between users of clinic services and OPSS, meaning that there may have been some double-counting of testing activity and diagnoses for individuals using both and also that successful treatment was assumed following transfer from OPSS to clinic-based care but not confirmed. The outcome of ‘rates of partner notification following an STI diagnosis’ was dropped because of a lack of usable data from any of the study sites.

The provision of sexual and reproductive healthcare in the UK is now fragmented and highly variable between regions in terms of available pathways for service users. It is therefore important to evaluate how outcomes have changed in association with the expansion of OPSS testing and remote clinical consultation services within specific areas. There was variation between our study areas in their implementation of remote care and in healthcare outcomes, but we did not find strong evidence that these changes were associated with increases in health inequity.

## Supplementary material

10.1136/bmjph-2025-004787 online supplemental file 1

10.1136/bmjph-2025-004787 online supplemental file 2

10.1136/bmjph-2025-004787 online supplemental file 3

## Data Availability

The datasets analysed for this observational study are not publicly available due to the sensitive data captured within them and the ethical approval in place for this study. Queries can be directed to the corresponding author.
