# Supplementary material for: Evaluating the impact of remote care in sexual and reproductive health services on outcomes and health inequalities in the UK: a retrospective observational study of routine service-level data
Source: BMJ Public Health. 2026 Sep 8;4(3):e004787. doi: 10.1136/bmjph-2025-004787 (PMC13560956; doi:10.1136/bmjph-2025-004787)
Supplement: Supplementary file 1 [file bmjph-4-3-s001.docx]

**Supplementary Appendix**

**Further methodological details**

*Sample size considerations*

The case study areas are each large enough to provide good power to detect moderate interactions between time periods and population subgroups (change in inequality) for the primary outcomes. Taking chlamydia treatment as an example, based on preliminary data we estimate 4000+ chlamydia cases in the face-to-face period, and 2000+ in the recent mixed mode period, and around 60% of cases were treated from clinic prescriptions in the first period and 50% in the recent period. For illustration we consider subgroups to be unassociated with treatment in the first period, and equally prevalent in the two time periods. The sample size provides good power for subgroups as small as 20% of chlamydia cases, which may be compared to either all other cases (80%), or to another subgroup representing half of all cases, or a different 20%. For these comparisons the sample size provides 80% power to detect an interaction odds ratio of 1.48, 1.51, and 1.64 respectively, corresponding to differences between subgroups in treatment rate of 10%, 10% and 12% respectively.

*Definition of contraceptive care*

Attendance records were coded as relating to any contraceptive service activity based on presence of any of the following within each episode of care: change in contraceptive method or status, SRHAD code relating to contraceptive use or implant removal, prescription of contraceptive pill or device, SHAPPT code for contraceptive or family planning advice.

*Missing data and multiple imputation*

Initial inspection of the data revealed that of the main patient characteristics defined for the analysis, substantial missing data (i.e. >5%) was present for ethnicity and IMD. For some sites there were also trends in the level of missing data for ethnicity over time, making it challenging to evaluate the impact of the introduction of OPSS according to ethnicity. To address this issue, we first considered multilevel multiple imputation (e.g. using the *jomo* package in R). However, this was not found to be feasible given the size and complexity of the datasets under investigation. We therefore conducted multiple imputation within Stata using only person-level covariables and then combined the results with the repeat episode of care observations for each person. Within the imputation process, data relating to individual test records were summarised for each individual as: any use of LARC, any positive chlamydia test, any chlamydia treatment, any positive gonorrhoea test, any gonorrhoea treatment, any indication of being a vulnerable service user, proportional use of remote care as initial contact, average date of service use.

Multiple imputation processes were run separately for each site, with the resulting set of imputed datasets used for analyses of all outcomes at each site, and included main covariables as in the statistical analyses (e.g. sex/sexuality group, age group, ethnicity, IMD quintile). We created 10 imputed datasets in each instance. Imputations were stratified by clinic and OPSS data for CSA-B.
