# Supplementary material for: Evaluating the impact of remote care in sexual and reproductive health services on outcomes and health inequalities in the UK: a retrospective observational study of routine service-level data
Source: BMJ Public Health. 2026 Sep 8;4(3):e004787. doi: 10.1136/bmjph-2025-004787 (PMC13560956; doi:10.1136/bmjph-2025-004787)
Supplement: Supplementary file 2 [file bmjph-4-3-s002.docx]

**Table S1:** Reference population data (ONS-2019 for IMD and Census 2021 for other variables) for each of the case study areas.

|  | CSA-A | CSA-B | CSA-C |
| --- | --- | --- | --- |
|  | **Census pop.**; n (%) | **Census pop.**; n (%) | **Census pop.**; n (%) |
| Overall | 1064140 | 886930 | 319414 |
| Sex grouping |  |  |  |
| Women | 550762 (51.8) | 467191 (52.7) | 165069 (51.7) |
| MSEW | 499294 (46.9) | 393863 (44.4) | 150769 (47.2) |
| MSM | 14071 (1.3) | 25840 (2.9) | 3590 (1.1) |
| Age |  |  |  |
| 16-24 | 183629 (17.3) | 126244 (14.2) | 37620 (11.8) |
| 25-34 | 192450 (18.1) | 206711 (23.3) | 40685 (12.7) |
| 35-49 | 262636 (24.7) | 243115 (27.4) | 61411 (19.2) |
| ≥50 | 425425 (40.0) | 310860 (35.0) | 179698 (56.3) |
| Ethnicity |  |  |  |
| Missing | - | - | - |
| White – British/Irish | 574827 (54.0) | 344308 (38.8) | 302317 (94.6) |
| White – other | 44943 (4.2) | 194637 (21.9) | 9140 (2.9) |
| Black African | 45997 (4.3) | 61446 (6.9) | 497 (0.2) |
| Black Carib. or other | 49857 (4.7) | 36210 (4.1) | 353 (0.1) |
| Asian | 275428 (25.9) | 131097 (14.8) | 3713 (1.2) |
| Any other | 38892 (3.7) | 75013 (8.5) | 1115 (0.3) |
| Mixed ethnicity | 34196 (3.2) | 44219 (5.0) | 2279 (0.7) |
| IMD |  |  |  |
| Missing | - | - | - |
| First quintile [most deprived] | 516875 (48.9) | 162543 (17.1) | 26988 (8.4) |
| Second quintile | 208830 (19.8) | 297466 (31.2) | 64574 (20.1) |
| Third quintile | 154039 (14.6) | 227665 (23.9) | 112947 (35.1) |
| Fourth quintile | 81166 (7.7) | 171142 (18.0) | 97019 (30.2) |
| Fifth quintile | 95945 (9.1) | 94034 (9.9) | 20162 (6.3) |

**Table S2:** Detailed information regarding sex, gender and sexuality (as available) for sexual and reproductive health service (SRHS) users in each year included in statistical analyses. SRHS users included based on any record of clinic attendance of self-sampling testing within each year. For CSA-B and CSA-C, users of both clinic and OPSS services in any given year may be double-counted as we do not have linkage between datasets.

|  | CSA-A | | CSA-B | | CSA-C | |
| --- | --- | --- | --- | --- | --- | --- |
|  | **2019;** n (%) | **2022**; n (%) | **2019**; n (%) | **2022**; n (%) | **2019**; n (%) | **2022**; n (%) |
| Clinic testers |  |  |  |  |  |  |
| Hetero. cis-man | 1112 (1.5) | 2829 (5.7) | 23388 (24.3) | 18619 (20.3) | 2525 (17.2) | 2056 (13.8) |
| Bi. cis-man | 220 (0.3) | 444 (0.9) | 1838 (1.9) | 2131 (2.3) | 122 (0.8) | 52 (0.4) |
| Homo. cis-man | 5535 (7.6) | 3811 (7.7) | 13517 (14.0) | 14185 (15.5) | 299 (2.0) | 599 (4.0) |
| Cis-man (unk. sexual.) | 19641 (27.0) | 12227 (24.8) | 907 (0.9) | 6339 (6.9) | 389 (2.6) | 439 (3.0) |
| Hetero. cis-woman | 2558 (3.5) | 4317 (8.7) | 52232 (54.2) | 43712 (47.7) | 9231 (62.8) | 9104 (61.3) |
| Bi. cis-woman | 125 (0.2) | 325 (0.7) | 2184 (2.3) | 3082 (3.4) | 74 (0.5) | 19 (0.1) |
| Homo. cis-woman | 23 (0.0) | 45 (0.1) | 487 (0.5) | 777 (0.8) | 50 (0.3) | 30 (0.2) |
| Cis-woman (unk. sexual.) | 43266 (59.6) | 25139 (50.9) | 1569 (1.6) | 2086 (2.3) | 2003 (13.6) | 2470 (16.6) |
| Transman | 17 (0.0) | 12 (0.0) | 26 (0.0) | 42 (0.0) | 2 (0.0) | 8 (0.1) |
| Transwoman | 26 (0.0) | 21 (0.0) | 22 (0.0) | 82 (0.1) | 0 (0.0) | 3 (0.0) |
| Transgender (unk. sex.) | 39 (0.1) | 47 (0.1) | 15 (0.0) | 0 (0.0) | 0 (0.0) | 13 (0.1) |
| Transgender or non-binary | 0 (0.0) | 0 (0.0) | 0 (0.0) | 0 (0.0) | 0 (0.0) | 0 (0.0) |
| Non-binary | 2 (0.0) | 51 (0.1) | 62 (0.1) | 446 (0.5) | 3 (0.0) | 17 (0.1) |
| No info. | 71 (0.1) | 109 (0.2) | 15 (0.0) | 19 (0.0) | 0 (0.0) | 47 (0.3) |

**Table S3:** Proportion of new Chlamydia diagnoses receiving treatment within 6 weeks of diagnosis, comparing the ‘mainly face-to face’ (2019) period with ‘mixed consultation model’ (2022) period. Change is estimated within categories (RR; 2022 *vs* 2019) along with relative change compared to reference category (aOR). This is the same as Table 3 in the main paper, with the addition of multivariable models.

|  | CSA-A | | | CSA-B | | | CSA-C | | |
| --- | --- | --- | --- | --- | --- | --- | --- | --- | --- |
|  | **2019;** n/N (%) | **2022;** n/N (%) (RR, 95%CI) | **2022**; aOR* (95%CI) | **2019;** n/N (%) | **2022;** n/N (%) (RR, 95%CI) | **2022**; aOR* (95%CI) | **2019;** n/N (%) | **2022;** n/N (%) (RR, 95%CI) | **2022**; aOR* (95%CI) |
| Overall outcome | 4833/6540 (73.9) | 3263/4634 (70.4) (0.95, 0.93-0.98) | 1.0 (0.8-1.3) | 4579/5878 (77.9) | 4297/4928 (87.2) (1.12, 1.10-1.14) | 3.8 (2.6-5.7) | 564/657 (85.8) | 535/580 (92.2) (1.07, 1.03-1.12) | 13.0 (1.9-89.7) |
| Outcome by group |  |  |  |  |  |  |  |  |  |
| F2F initial | 3373/3927 (85.9) | 1748/2158 (81.0) |  | 3397/4669 (72.8) | 1058/1514 (69.9) |  | 519/594 (87.4) | 65/71 (91.5) |  |
| OPSS | 1436/2583 (55.6) | 1099/1969 (55.8) |  | 1182/1209 (97.8) | 3107/3228 (96.3) |  | 45/63 (71.4) | 396/426 (93.0) |  |
| RCC | 24/30 (80.0) | 416/507 (82.1) |  |  | 132/186 (71.0) |  |  | 74/83 (89.2) |  |
| Sex grouping *[missing, eq. P]* | 0.1% | 0.2%, p<0.001 |  | 0.1% | 0.1%, 0.018 |  | 0.0% | 0.0%, 0.131 |  |
| Women | 2680/3797 (70.6) | 1748/2528 (69.1) (0.98, 0.95-1.01) | Ref. | 1887/2390 (79.0) | 1766/1948 (90.7) (1.15, 1.12-1.18) | Ref. | 309/371 (83.3) | 317/343 (92.4) (1.11, 1.05-1.17) | Ref. |
| MSEW | 1616/2052 (78.8) | 1035/1494 (69.3) (0.88, 0.84-0.92) | 0.7 (0.5-0.8) | 1242/1582 (78.5) | 1097/1236 (88.8) (1.13, 1.09-1.17) | 0.9 (0.7-1.2) | 226/249 (90.8) | 166/178 (93.3) (1.03, 0.97-1.09) | 0.5 (0.2-1.3) |
| MSM | 537/691 (77.7) | 480/612 (78.4) (1.01, 0.95-1.07) | 1.0 (0.8-1.4) | 1450/1906 (76.1) | 1434/1744 (82.2) (1.08, 1.05-1.12) | 0.7 (0.5-0.9) | 29/37 (78.4) | 52/59 (88.1) (1.12, 0.93-1.36) | 1.1 (0.2-5.6) |
| Age *[missing, eq. P]* | 0.0% | 0.0%, p=0.581 |  | 0.0% | 0.0%, 0.001 |  | 0.0% | 0.0%, 0.312 |  |
| 16-24 | 2946/3997 (73.7) | 1894/2737 (69.2) (0.94, 0.91-0.97) | 0.9 (0.7-1.1) | 1649/2104 (78.4) | 1566/1747 (89.6) (1.14, 1.11-1.18) | 1.0 (0.7-1.2) | 392/448 (87.5) | 374/403 (92.8) (1.06, 1.02-1.11) | 0.5 (0.2-1.5) |
| 25-34 | 1353/1844 (73.4) | 926/1297 (71.4) (0.97, 0.93-1.02) | Ref. | 1822/2326 (78.3) | 1733/1947 (89.0) (1.14, 1.11-1.17) | Ref. | 131/160 (81.9) | 116/123 (94.3) (1.15, 1.06-1.26) | Ref. |
| 35-49 | 427/563 (75.8) | 367/501 (73.3) (0.97, 0.90-1.04) | 1.0 (0.7-1.3) | 875/1138 (76.9) | 797/952 (83.7) (1.09, 1.04-1.14) | 0.8 (0.6-1.0) | 32/38 (84.2) | 35/42 (83.3) (0.99, 0.82-1.20) | 0.3 (0.0-1.3) |
| ≥50 | 107/136 (78.7) | 76/99 (76.8) (0.98, 0.85-1.12) | 0.9 (0.5-1.8) | 233/310 (75.2) | 201/282 (71.3) (0.95, 0.86-1.04) | 0.4 (0.3-0.6) | 9/11 (81.8) | 10/12 (83.3) (1.02, 0.71-1.46) | 0.4 (0.0-5.1) |
| Ethnicity *[missing, eq. P]* | 7.1% | 15.2%, p=0.372 |  | 27.8% | 8.5%, 0.774 |  | 0.8% | 22.2%, 0.932 |  |
| White - Br/Ir | 66.2 | 64.1 (0.97, 0.93-1.01) | Ref. | 78.4 | 89.6 (1.14, 1.10-1.18) | Ref. | 85.7 | 92.2 (1.08, 1.03-1.12) | Ref. |
| White - other | 76.6 | 74.0 (0.97, 0.87-1.07) | 0.9 (0.6-1.4) | 78.8 | 85.7 (1.09, 1.04-1.14) | 0.7 (0.5-1.1) | 88.9 | 93.8 (1.05, 0.87-1.28) | 0.8 (0.1-9.9) |
| Black African | 80.6 | 76.1 (0.94, 0.88-1.01) | 0.9 (0.7-1.4) | 77.7 | 87.8 (1.13, 1.05-1.22) | 0.7 (0.4-1.2) | - | - |  |
| Black Carib/oth | 84.8 | 76.1 (0.90, 0.86-0.94) | 0.7 (0.5-0.9) | 78.8 | 87.0 (1.10, 1.04-1.18) | 0.6 (0.4-1.1) | - | - |  |
| Asian | 79.2 | 73.3 (0.93, 0.86-0.99) | 0.8 (0.6-1.1) | 74.9 | 82.7 (1.10, 1.02-1.19) | 0.7 (0.4-1.1) | - | - |  |
| Any other | 78.6 | 74.9 (0.95, 0.77-1.17) | 0.9 (0.4-2.2) | 80.5 | 89.7 (1.12, 1.01-1.24) | 0.9 (0.4-1.8) | 88.9 | 92.1 (1.04, 0.83-1.30) | 0.6 (0.0-7.8) |
| Mixed | 76.4 | 74.3 (0.97, 0.91-1.04) | 1.0 (0.7-1.4) | 75.0 | 83.8 (1.12, 1.05-1.19) | 0.6 (0.4-1.0) | - | - |  |
| IMD *[missing, eq. P]* | 5.9% | 8.1%, p=0.106 |  | 0.8% | 0.4%, 0.323 |  | 9.0% | 9.1%, 0.470 |  |
| First quintile [most deprived] | 76.9 | 71.6 (0.93, 0.90-0.96) | Ref. | 76.1 | 86.9 (1.14, 1.10-1.19) | Ref. | 83.1 | 95.4 (1.15, 1.04-1.26) | Ref. |
| Second quintile | 71.8 | 72.3 (1.01, 0.96-1.06) | 1.3 (1.0-1.6) | 79.4 | 87.7 (1.11, 1.08-1.14) | 0.9 (0.7-1.2) | 86.2 | 89.7 (1.04, 0.95-1.14) | 0.2 (0.0-1.2) |
| Third quintile | 69.1 | 68.3 (0.99, 0.92-1.06) | 1.2 (0.9-1.6) | 78.1 | 87.9 (1.13, 1.09-1.17) | 1.0 (0.7-1.4) | 87.2 | 92.9 (1.07, 1.00-1.14) | 0.3 (0.1-1.7) |
| Fourth quintile | 66.8 | 64.2 (0.96, 0.85-1.09) | 1.1 (0.7-1.6) | 79.2 | 85.9 (1.08, 1.03-1.14) | 0.7 (0.5-1.1) | 84.7 | 92.6 (1.09, 1.01-1.18) | 0.5 (0.1-2.3) |
| Fifth quintile | 68.4 | 61.8 (0.90, 0.78-1.04) | 0.9 (0.6-1.5) | 71.3 | 83.9 (1.18, 1.08-1.28) | 1.0 (0.6-1.6) | 90.3 | 89.0 (0.99, 0.84-1.16) | 0.2 (0.0-1.4) |

aOR, adjusted odds ratio; eq. P, ‘interaction’ P-value testing equality of RR for change over time across groups; IMD, index of multiple deprivation; MSEW, men who have sex exclusively with women; MSM, men who have sex with men; OPSS, online postal self-sampling; RCC, remote telephone or video clinical consultation; RR, relative risk. % and RR values calculated using multiple imputation for ethnicity and IMD values, with other missing data excluded. *With adjustment for all variables listed in table; The ‘overall’ aOR value (note not aRR) represents a comparison to the baseline year for a 25-34 year-old woman of white British/Irish ethnicity living in an area with IMD in the first quintile, for other demographic variables such as age the aOR values represent how much greater is the change from baseline year in each group of the variable relative to the respective reference group.

**Table S4:** Proportion of new Chlamydia diagnoses receiving treatment within 3 weeks of diagnosis, comparing the ‘mainly face-to face’ (2019) period with ‘mixed consultation model’ (2022) period. Change is estimated within categories (RR; 2022 *vs* 2019) along with relative change compared to reference category (aOR).

|  | CSA-A | | | CSA-B | | | CSA-C | | |
| --- | --- | --- | --- | --- | --- | --- | --- | --- | --- |
|  | **2019;** n/N (%) | **2022;** n/N (%) (RR, 95%CI) | **2022**; aOR* (95%CI) | **2019;** n/N (%) | **2022;** n/N (%) (RR, 95%CI) | **2022**; aOR* (95%CI) | **2019;** n/N (%) | **2022;** n/N (%) (RR, 95%CI) | **2022**; aOR* (95%CI) |
| Overall outcome | 4618/6540 (70.6) | 3019/4634 (65.1) (0.92, 0.90-0.95) | 0.9 (0.7-1.1) | 4505/5878 (76.6) | 4266/4928 (86.6) (1.13, 1.11-1.15) | 3.7 (2.5-5.4) | 537/657 (81.7) | 530/580 (91.4) (1.12, 1.07-1.17) | 8.3 (1.8-38.2) |
| Outcome by group |  |  |  |  |  |  |  |  |  |
| F2F initial | 3270/3927 (83.3) | 1655/2158 (76.7) |  | 3327/4669 (71.3) | 1047/1514 (69.2) |  | 495/594 (83.3) | 65/71 (91.5) |  |
| OPSS | 1326/2583 (51.3) | 1004/1969 (51.0) |  | 1178/1209 (97.4) | 3087/3228 (95.6) |  | 42/63 (66.7) | 394/426 (92.5) |  |
| RCC | 22/30 (73.3) | 360/507 (71.0) |  |  | 132/186 (71.0) |  |  | 71/83 (85.5) |  |
| Sex grouping *[missing, eq. P]* | 0.1% | 0.2%, <0.001 |  | 0.1% | 0.1%, 0.065 |  | 0.0% | 0.0%, 0.055 |  |
| Women | 2545/3797 (67.0) | 1634/2528 (64.6) (0.96, 0.93-1.00) | Ref. | 1864/2390 (78.0) | 1755/1948 (90.1) (1.16, 1.13-1.19) | Ref. | 292/371 (78.7) | 316/343 (92.1) (1.17, 1.10-1.24) | Ref. |
| MSEW | 1565/2052 (76.3) | 951/1494 (63.7) (0.83, 0.80-0.87) | 0.6 (0.5-0.7) | 1224/1582 (77.4) | 1089/1236 (88.1) (1.14, 1.10-1.18) | 0.9 (0.7-1.2) | 218/249 (87.6) | 164/178 (92.1) (1.05, 0.99-1.12) | 0.5 (0.2-1.2) |
| MSM | 508/691 (73.5) | 434/612 (70.9) (0.96, 0.90-1.03) | 0.9 (0.7-1.2) | 1417/1906 (74.3) | 1422/1744 (81.5) (1.10, 1.06-1.14) | 0.7 (0.5-0.9) | 27/37 (73.0) | 50/59 (84.7) (1.16, 0.92-1.47) | 0.7 (0.2-3.2) |
| Age *[missing, eq. P]* | 0.0% | 0.0%, 0.944 |  | 0.0% | 0.0%, 0.003 |  | 0.0% | 0.0%, 0.756 |  |
| 16-24 | 2801/3997 (70.1) | 1759/2737 (64.3) (0.92, 0.89-0.95) | 0.9 (0.8-1.1) | 1622/2104 (77.1) | 1554/1747 (89.0) (1.15, 1.12-1.19) | 1.0 (0.7-1.2) | 372/448 (83.0) | 371/403 (92.1) (1.11, 1.05-1.17) | 0.7 (0.3-1.7) |
| 25-34 | 1311/1844 (71.1) | 854/1297 (65.8) (0.93, 0.88-0.97) | Ref. | 1794/2326 (77.1) | 1722/1947 (88.4) (1.15, 1.12-1.18) | Ref. | 127/160 (79.4) | 114/123 (92.7) (1.17, 1.06-1.28) | Ref. |
| 35-49 | 405/563 (71.9) | 339/501 (67.7) (0.94, 0.87-1.02) | 1.1 (0.8-1.5) | 862/1138 (75.7) | 790/952 (83.0) (1.10, 1.05-1.15) | 0.7 (0.6-1.0) | 29/38 (76.3) | 35/42 (83.3) (1.09, 0.87-1.38) | 0.5 (0.1-2.3) |
| ≥50 | 101/136 (74.3) | 67/99 (67.7) (0.91, 0.77-1.07) | 0.9 (0.5-1.6) | 227/310 (73.2) | 200/282 (70.9) (0.97, 0.88-1.07) | 0.4 (0.3-0.6) | 9/11 (81.8) | 10/12 (83.3) (1.02, 0.71-1.46) | 0.5 (0.0-6.3) |
| Ethnicity *[missing, eq. P]* | 7.1% | 15.2%, 0.415 |  | 27.8% | 8.5%, 0.898 |  | 0.8% | 22.2%, 0.725 |  |
| White - Br/Ir | 63.1 | 58.4 (0.92, 0.88-0.97) | Ref. | 77.6 | 89.1 (1.15, 1.11-1.19) | Ref. | 81.6 | 91.3 (1.12, 1.07-1.17) | Ref. |
| White - other | 75.2 | 68.6 (0.91, 0.80-1.04) | 0.8 (0.5-1.4) | 77.2 | 85.0 (1.10, 1.06-1.15) | 0.7 (0.5-1.0) | 88.9 | 93.6 (1.05, 0.87-1.28) | 0.8 (0.1-9.8) |
| Black African | 76.5 | 71.5 (0.93, 0.87-1.00) | 1.1 (0.8-1.5) | 76.2 | 86.6 (1.14, 1.05-1.23) | 0.7 (0.5-1.2) |  |  |  |
| Black Carib/oth | 81.2 | 70.0 (0.86, 0.82-0.91) | 0.8 (0.6-1.0) | 76.2 | 86.4 (1.13, 1.05-1.23) | 0.7 (0.4-1.2) |  |  |  |
| Asian | 75.4 | 70.1 (0.93, 0.86-1.01) | 1.0 (0.7-1.4) | 73.2 | 82.5 (1.13, 1.04-1.23) | 0.7 (0.5-1.2) |  |  |  |
| Any other | 73.4 | 66.5 (0.91, 0.72-1.14) | 0.9 (0.4-2.0) | 79.6 | 89.7 (1.13, 1.03-1.24) | 0.9 (0.5-1.7) | 77.5 | 93.4 (1.20, 0.91-1.59) | 1.7 (0.2-18.2) |
| Mixed | 73.4 | 70.0 (0.95, 0.89-1.03) | 1.1 (0.8-1.5) | 74.4 | 82.9 (1.12, 1.05-1.19) | 0.6 (0.4-0.9) |  |  |  |
| IMD *[missing, eq. P]* | 5.9% | 8.1%, 0.101 |  | 0.8% | 0.4%, 0.218 |  | 9.0% | 9.1%, 0.624 |  |
| First quintile [most deprived] | 73.6 | 66.0 (0.90, 0.87-0.93) | Ref. | 74.5 | 86.3 (1.16, 1.11-1.21) | Ref. | 80.0 | 94.6 (1.18, 1.05-1.33) | Ref. |
| Second quintile | 68.8 | 66.4 (0.96, 0.91-1.02) | 1.2 (1.0-1.5) | 78.2 | 87.0 (1.11, 1.08-1.14) | 0.9 (0.6-1.2) | 82.6 | 88.0 (1.07, 0.96-1.18) | 0.3 (0.1-1.5) |
| Third quintile | 64.2 | 63.3 (0.98, 0.91-1.06) | 1.4 (1.1-1.7) | 76.8 | 87.5 (1.14, 1.10-1.18) | 1.0 (0.7-1.4) | 81.1 | 91.3 (1.13, 1.04-1.22) | 0.5 (0.1-2.1) |
| Fourth quintile | 64.6 | 61.3 (0.95, 0.83-1.08) | 1.2 (0.8-1.7) | 78.3 | 85.3 (1.09, 1.03-1.15) | 0.7 (0.5-1.0) | 81.6 | 92.6 (1.14, 1.05-1.23) | 0.7 (0.2-3.0) |
| Fifth quintile | 66.2 | 60.2 (0.91, 0.79-1.05) | 1.1 (0.7-1.7) | 70.0 | 83.2 (1.19, 1.09-1.30) | 1.0 (0.6-1.6) | 87.4 | 90.4 (1.03, 0.87-1.23) | 0.3 (0.0-2.0) |

aOR, adjusted odds ratio, OR; eq. P, ‘interaction’ P-value testing equality of RR across groups; RCC, remote telephone or video clinical consultation; RR, relative risk. % and RR values calculated using multiple imputation for ethnicity and IMD values, with other missing data excluded. *With adjustment for all variables listed in table; The ‘overall’ aOR value (note not aRR) represents a comparison to the baseline year for a 25-34 year-old woman of white British/Irish ethnicity living in an area with IMD in the first quintile, for other demographic variables such as age the aOR values represent how much greater is the change from baseline year in each group of the variable relative to the respective reference group.

**Table S5:** Proportion of new Chlamydia diagnoses receiving treatment within 6 weeks of diagnosis, comparing initial contact via remote care (RC) or face-to-face (F2F) within 2022.

|  | CSA-A | | | CSA-B | | | CSA-C | |
| --- | --- | --- | --- | --- | --- | --- | --- | --- |
|  | F2F; n/N (%) | RC; n/N (%) (RR, 95%CI) | RC; aOR (95%CI) | F2F; n/N (%) | RC; n/N (%) (RR, 95%CI) | RC; aOR (95%CI) | F2F; n/N (%) | RC; n/N (%) (RR, 95%CI) |
| Overall outcome | 1748/2158 (81.0) | 1515/2476 (61.2) (0.76, 0.73-0.78) | 0.3 (0.2-0.5) | 1058/1514 (69.9) | 3239/3414 (94.9) (1.36, 1.31-1.40) | 13.5 (6.8-26.6) | 65/71 (91.5) | 470/509 (92.3) (1.01, 0.94-1.09) |
| Outcome by group |  |  |  |  |  |  |  |  |
| OPSS | --- | 1099/1969 (55.8) |  | --- | 3107/3228 (96.3) |  | --- | 396/426 (93.0) |
| RCC | --- | 416/507 (82.1) |  | --- | 132/186 (71.0) |  | --- | 74/83 (89.2) |
| Sex grouping *[missing, eq. P]* | 0.2% | 0.2%, <0.001 |  | 0.3% | 0.0%, <0.001 |  | 0.0% | 0.0%, 0.654 |
| Women | 843/1017 (82.9) | 905/1511 (59.9) (0.72, 0.69-0.76) | Ref. | 292/405 (72.1) | 1474/1543 (95.5) (1.32, 1.25-1.41) | Ref. | 34/37 (91.9) | 283/306 (92.5) (1.01, 0.91-1.11) |
| MSEW | 688/867 (79.4) | 347/627 (55.3) (0.70, 0.64-0.75) | 1.0 (0.7-1.4) | 309/400 (77.2) | 788/836 (94.3) (1.22, 1.15-1.29) | 0.6 (0.4-1.0) | 18/18 (100.0) | 148/160 (92.5) (1.00, 1.00-1.00) |
| MSM | 217/274 (79.2) | 263/338 (77.8) (0.98, 0.91-1.07) | 3.0 (1.9-4.9) | 457/709 (64.5) | 977/1035 (94.4) (1.46, 1.38-1.55) | 1.0 (0.6-1.6) | 13/16 (81.2) | 39/43 (90.7) (1.12, 0.88-1.41) |
| Age *[missing, eq. P]* | 0.0% | 0.0%, 0.058 |  | 0.0% | 0.0%, 0.029 |  |  |  |
| 16-24 | 1003/1233 (81.3) | 891/1504 (59.2) (0.73, 0.69-0.77) | 1.0 (0.7-1.4) | 291/395 (73.7) | 1275/1352 (94.3) (1.28, 1.20-1.36) | 0.7 (0.4-1.1) |  |  |
| 25-34 | 498/617 (80.7) | 428/680 (62.9) (0.78, 0.73-0.84) | Ref. | 362/512 (70.7) | 1371/1435 (95.5) (1.35, 1.28-1.43) | Ref. |  |  |
| 35-49 | 203/254 (79.9) | 164/247 (66.4) (0.83, 0.75-0.93) | 1.0 (0.6-1.7) | 297/424 (70.0) | 500/528 (94.7) (1.35, 1.27-1.44) | 0.8 (0.5-1.4) |  |  |
| ≥50 | 44/54 (81.5) | 32/45 (71.1) (0.78, 0.73-0.84) | 1.0 (0.4-2.7) | 108/183 (59.0) | 93/99 (93.9) (1.35, 1.28-1.43) | 1.0 (0.4-2.6) |  |  |
| Ethnicity *[missing, eq. P]* | 20.2% | 11.0%, 0.113 |  | 24.2% | 1.6%, 0.722 |  |  |  |
| White - Br/Ir | 78.4 | 56.5 (0.72, 0.67-0.77) | Ref. | 70.2 | 96.1 (1.37, 1.28-1.47) | Ref. |  |  |
| White - other | 87.0 | 63.6 (0.73, 0.61-0.87) | 0.6 (0.2-1.5) | 67.4 | 94.4 (1.40, 1.30-1.51) | 0.8 (0.5-1.4) |  |  |
| Black African | 81.4 | 65.9 (0.81, 0.72-0.91) | 1.2 (0.7-2.1) | 75.6 | 94.7 (1.25, 1.14-1.38) | 0.7 (0.3-1.5) |  |  |
| Black Carib/oth | 81.9 | 68.9 (0.84, 0.77-0.92) | 1.3 (0.9-2.0) | 71.0 | 95.3 (1.34, 1.17-1.54) | 1.0 (0.4-2.4) |  |  |
| Asian | 80.1 | 66.8 (0.83, 0.74-0.94) | 1.2 (0.7-2.0) | 71.1 | 91.8 (1.29, 1.14-1.46) | 0.4 (0.2-0.9) |  |  |
| Any other | 79.8 | 68.6 (0.86, 0.62-1.19) | 1.3 (0.3-5.1) | 68.1 | 95.6 (1.41, 1.14-1.74) | 1.1 (0.4-2.8) |  |  |
| Mixed | 83.7 | 65.1 (0.78, 0.70-0.87) | 1.0 (0.6-1.6) | 67.8 | 92.5 (1.36, 1.22-1.52) | 0.6 (0.3-1.2) |  |  |
| IMD *[missing, eq. P]* | 14.3% | 2.8%, 0.249 |  | 1.2% | 0.1%, 0.525 |  |  |  |
| First quintile [most deprived] | 80.1 | 62.5 (0.78, 0.74-0.82) | Ref. | 69.5 | 94.9 (1.37, 1.27-1.47) | Ref. |  |  |
| Second quintile | 82.9 | 62.9 (0.76, 0.70-0.82) | 0.8 (0.6-1.2) | 71.5 | 94.6 (1.32, 1.25-1.40) | 0.8 (0.5-1.3) |  |  |
| Third quintile | 83.9 | 60.5 (0.72, 0.65-0.80) | 0.8 (0.5-1.2) | 71.1 | 95.5 (1.34, 1.25-1.44) | 1.0 (0.5-1.8) |  |  |
| Fourth quintile | 81.1 | 54.0 (0.67, 0.56-0.79) | 0.6 (0.3-1.2) | 64.5 | 95.5 (1.48, 1.32-1.66) | 1.4 (0.7-2.8) |  |  |
| Fifth quintile | 76.8 | 51.2 (0.67, 0.53-0.84) | 0.8 (0.4-1.7) | 67.2 | 92.4 (1.37, 1.17-1.61) | 0.7 (0.3-1.6) |  |  |

aOR, adjusted odds ratio, OR; eq. P, ‘interaction’ P-value testing equality of RR across groups; RCC, remote telephone or video clinical consultation; RR, relative risk. % and RR values calculated using multiple imputation for ethnicity and IMD values, with other missing data excluded. *With adjustment for all variables listed in table; The ‘overall’ aOR value (note not aRR) represents a comparison to the baseline year for a 25-34 year-old woman of white British/Irish ethnicity living in an area with IMD in the first quintile, for other demographic variables such as age the aOR values represent how much greater is the change from baseline year in each group of the variable relative to the respective reference group.

**Table S6:** Proportion of new gonorrhoea diagnoses receiving treatment within 6 weeks of diagnosis, comparing the ‘mainly face-to face’ (2019) period with ‘mixed consultation model’ (2022) period. Change is estimated within categories (RR; 2022 *vs* 2019) along with relative change compared to reference category (aOR).

|  | CSA-A | | | CSA-B | | | CSA-C | |
| --- | --- | --- | --- | --- | --- | --- | --- | --- |
|  | **2019;** n/N (%) | **2022;** n/N (%) (RR, 95%CI) | **2022**; aOR* (95%CI) | **2019;** n/N (%) | **2022;** n/N (%) (RR, 95%CI) | **2022**; aOR* (95%CI) | **2019;** n/N (%) | **2022;** n/N (%) (RR, 95%CI) |
| Overall outcome | 2242/2670 (84.0) | 2170/2617 (82.9) (0.99, 0.96-1.01) | 0.7 (0.4-1.0) | 1680/2104 (79.8) | 2102/2426 (86.6) (1.09, 1.06-1.11) | 1.9 (1.1-3.3) | 80/99 (80.8) | 118/134 (88.1) (1.09, 0.97-1.22) |
| Outcome by group |  |  |  |  |  |  |  |  |
| F2F initial | 1743/1964 (88.7) | 1375/1549 (88.8) |  | 1276/1681 (75.9) | 665/890 (74.7) |  | 69/82 (84.1) | 24/25 (96.0) |
| OPSS | 478/680 (70.3) | 478/677 (70.6) |  | 404/423 (95.5) | 1393/1479 (94.2) |  | 11/17 (64.7) | 70/83 (84.3) |
| RCC | 21/26 (80.8) | 317/391 (81.1) |  |  | 44/57 (77.2) |  |  | 24/26 (92.3) |
| Sex grouping *[missing, eq. P]* | 0.4% | 0.6%, 0.028 |  | 0.0% | 0.1%, 0.980 |  | 0.0% | 0.0%, 0.070 |
| Women | 852/1042 (81.8) | 732/951 (77.0) (0.94, 0.90-0.99) | Ref. | 367/460 (79.8) | 431/498 (86.5) (1.08, 1.02-1.15) | Ref. | 37/43 (86.0) | 53/64 (82.8) (0.96, 0.82-1.14) |
| MSEW | 670/763 (87.8) | 712/812 (87.7) (1.00, 0.96-1.04) | 1.4 (0.9-2.1) | 322/418 (77.0) | 339/404 (83.9) (1.09, 1.02-1.17) | 1.0 (0.6-1.7) | 16/19 (84.2) | 30/33 (90.9) (1.08, 0.86-1.35) |
| MSM | 720/865 (83.2) | 726/854 (85.0) (1.02, 0.98-1.06) | 1.7 (1.2-2.5) | 991/1226 (80.8) | 1332/1524 (87.4) (1.08, 1.05-1.12) | 0.9 (0.6-1.4) | 27/37 (73.0) | 35/37 (94.6) (1.30, 1.07-1.57) |
| Age *[missing, eq. P]* | 0.0% | 0.0%, 0.323 |  | 0.0% | 0.0%, 0.146 |  |  |  |
| 16-24 | 973/1203 (80.9) | 923/1141 (80.9) (1.00, 0.96-1.04) | 1.5 (1.0-2.1) | 441/548 (80.5) | 489/575 (85.0) (1.06, 1.00-1.12) | 0.8 (0.6-1.3) |  |  |
| 25-34 | 780/900 (86.7) | 736/880 (83.6) (0.97, 0.93-1.00) | Ref. | 690/852 (81.0) | 923/1050 (87.9) (1.09, 1.04-1.13) | Ref. |  |  |
| 35-49 | 376/430 (87.4) | 404/473 (85.4) (0.98, 0.93-1.03) | 1.0 (0.6-1.7) | 420/541 (77.6) | 546/620 (88.1) (1.13, 1.07-1.20) | 1.3 (0.9-2.0) |  |  |
| ≥50 | 113/137 (82.5) | 107/123 (87.0) (1.05, 0.95-1.17) | 1.8 (0.9-3.9) | 129/163 (79.1) | 144/181 (79.6) (1.01, 0.90-1.12) | 0.7 (0.4-1.2) |  |  |
| Ethnicity *[missing, eq. P]* | 8.3% | 17.6%, 0.106 |  | 27.4% | 9.6%, 0.743 |  |  |  |
| White - Br/Ir | 82.4 | 81.9 (0.99, 0.96-1.03) | Ref. | 81.6 | 87.0 (1.07, 1.02-1.11) | Ref. |  |  |
| White - other | 86.1 | 76.3 (0.89, 0.78-1.01) | 0.5 (0.2-1.0) | 81.1 | 89.9 (1.11, 1.05-1.17) | 1.3 (0.8-2.1) |  |  |
| Black African | 78.3 | 82.8 (1.06, 0.96-1.17) | 1.3 (0.7-2.4) | 73.0 | 75.7 (1.04, 0.89-1.20) | 0.7 (0.4-1.3) |  |  |
| Black Carib/oth | 88.9 | 83.2 (0.94, 0.89-0.99) | 0.6 (0.4-0.9) | 79.2 | 80.8 (1.02, 0.92-1.13) | 0.7 (0.4-1.3) |  |  |
| Asian | 85.4 | 88.0 (1.03, 0.96-1.10) | 1.2 (0.7-2.2) | 77.4 | 88.4 (1.14, 1.00-1.31) | 1.4 (0.6-3.2) |  |  |
| Any other | 78.9 | 82.0 (1.04, 0.82-1.31) | 1.1 (0.3-3.8) | 83.2 | 89.8 (1.08, 0.96-1.22) | 1.1 (0.4-2.7) |  |  |
| Mixed | 83.6 | 83.6 (1.00, 0.93-1.08) | 1.0 (0.6-1.7) | 75.8 | 84.1 (1.11, 1.00-1.23) | 1.1 (0.6-1.9) |  |  |
| IMD *[missing, eq. P]* | 6.0% | 7.4%, 0.742 |  | 0.9% | 0.6%, 0.103 |  |  |  |
| First quintile [most deprived] | 84.7 | 84.4 (1.00, 0.97-1.03) | Ref. | 75.2 | 84.1 (1.12, 1.05-1.20) | Ref. |  |  |
| Second quintile | 83.2 | 83.0 (1.00, 0.94-1.06) | 1.0 (0.7-1.5) | 80.9 | 88.5 (1.09, 1.05-1.14) | 1.0 (0.7-1.6) |  |  |
| Third quintile | 83.0 | 79.9 (0.96, 0.89-1.04) | 0.8 (0.5-1.2) | 81.7 | 85.5 (1.05, 0.99-1.11) | 0.7 (0.4-1.2) |  |  |
| Fourth quintile | 83.4 | 77.3 (0.93, 0.82-1.05) | 0.6 (0.3-1.3) | 84.0 | 86.3 (1.03, 0.96-1.10) | 0.6 (0.4-1.2) |  |  |
| Fifth quintile | 80.7 | 78.3 (0.97, 0.84-1.12) | 0.8 (0.3-1.7) | 72.1 | 88.8 (1.23, 1.07-1.42) | 1.6 (0.7-3.8) |  |  |

aOR, adjusted odds ratio, OR; eq. P, ‘interaction’ P-value testing equality of RR across groups; RCC, remote telephone or video clinical consultation; RR, relative risk. % and RR values calculated using multiple imputation for ethnicity and IMD values, with other missing data excluded. *With adjustment for all variables listed in table; The ‘overall’ aOR value (note not aRR) represents a comparison to the baseline year for a 25-34 year-old woman of white British/Irish ethnicity living in an area with IMD in the first quintile, for other demographic variables such as age the aOR values represent how much greater is the change from baseline year in each group of the variable relative to the respective reference group.

**Table S7:** Proportion of new gonorrhoea diagnoses receiving treatment within 6 weeks of diagnosis, comparing initial contact via remote care (RC) or face-to-face (F2F) within 2022.

|  | CSA-A | | | CSA-B | | CSA-C | |
| --- | --- | --- | --- | --- | --- | --- | --- |
|  | F2F; n/N (%) | RC; n/N (%) (RR, 95%CI) | RC; aOR (95%CI) | F2F; n/N (%) | RC; n/N (%) (RR, 95%CI) | F2F; n/N (%) | RC; n/N (%) (RR, 95%CI) |
| Overall outcome | 1375/1549 (88.8) | 795/1068 (74.4) (0.84, 0.81-0.87) | 0.6 (0.3-1.1) | 665/890 (74.7) | 1437/1536 (93.6) (1.25, 1.20-1.30) | 24/25 (96.0) | 94/109 (86.2) (0.90, 0.80-1.00) |
| Outcome by group |  |  |  |  |  |  |  |
| OPSS | --- | 478/677 (70.6) |  | --- | 1393/1479 (94.2) | --- | 70/83 (84.3) |
| RCC | --- | 317/391 (81.1) |  | --- | 44/57 (77.2) | --- | 24/26 (92.3) |
| Sex grouping *[missing, eq. P]* | 0.6% | 0.7%, <0.001 |  | 0.2% | 0.1%, <0.001 |  |  |
| Women | 364/440 (82.7) | 368/511 (72.0) (0.87, 0.81-0.93) | Ref. | 132/175 (75.4) | 299/323 (92.6) (1.23, 1.12-1.34) |  |  |
| MSEW | 601/644 (93.3) | 111/168 (66.1) (0.71, 0.63-0.79) | 0.3 (0.1-0.5) | 201/248 (81.0) | 138/156 (88.5) (1.09, 1.01-1.19) |  |  |
| MSM | 410/465 (88.2) | 316/389 (81.2) (0.92, 0.87-0.98) | 1.3 (0.7-2.1) | 332/467 (71.1) | 1000/1057 (94.6) (1.33, 1.25-1.41) |  |  |
| Age *[missing, eq. P]* | 0.0% | 0.0%, 0.139 |  | 0.0% | 0.0%, 0.242 |  |  |
| 16-24 | 582/665 (87.5) | 341/476 (71.6) (0.82, 0.77-0.87) | 0.8 (0.5-1.3) | 161/213 (75.6) | 328/362 (90.6) (1.20, 1.10-1.30) |  |  |
| 25-34 | 447/510 (87.6) | 289/370 (78.1) (0.89, 0.84-0.95) | Ref. | 246/330 (74.5) | 677/720 (94.0) (1.26, 1.18-1.35) |  |  |
| 35-49 | 274/299 (91.6) | 130/174 (74.7) (0.82, 0.74-0.89) | 0.6 (0.3-1.1) | 193/249 (77.5) | 353/371 (95.1) (1.23, 1.15-1.32) |  |  |
| ≥50 | 72/75 (96.0) | 35/48 (72.9) (0.89, 0.84-0.95) | 0.2 (0.0-0.6) | 65/98 (66.3) | 79/83 (95.2) (1.26, 1.18-1.35) |  |  |
| Ethnicity *[missing, eq. P]* | 21.4% | 12.2%, 0.268 |  |  |  |  |  |
| White - Br/Ir | 88.6 | 75.8 (0.86, 0.81-0.91) | Ref. |  |  |  |  |
| White - other | 89.0 | 58.6 (0.66, 0.51-0.85) | 0.4 (0.1-1.3) |  |  |  |  |
| Black African | 88.4 | 64.4 (0.73, 0.59-0.90) | 0.9 (0.4-2.3) |  |  |  |  |
| Black Carib/oth | 87.8 | 72.4 (0.82, 0.74-0.92) | 1.4 (0.7-2.7) |  |  |  |  |
| Asian | 91.1 | 81.9 (0.90, 0.80-1.01) | 1.2 (0.5-3.1) |  |  |  |  |
| Any other | 83.0 | 80.7 (0.97, 0.71-1.32) | 2.8 (0.5-16.9) |  |  |  |  |
| Mixed | 89.2 | 73.8 (0.83, 0.73-0.94) | 1.0 (0.5-2.1) |  |  |  |  |
| IMD *[missing, eq. P]* | 10.5% | 2.9%, 0.880 |  |  |  |  |  |
| First quintile [most deprived] | 90.0 | 74.7 (0.83, 0.79-0.88) | Ref. |  |  |  |  |
| Second quintile | 88.2 | 76.5 (0.87, 0.80-0.95) | 1.2 (0.6-2.1) |  |  |  |  |
| Third quintile | 86.1 | 74.5 (0.87, 0.78-0.96) | 1.3 (0.6-2.5) |  |  |  |  |
| Fourth quintile | 85.3 | 68.8 (0.81, 0.66-0.98) | 0.9 (0.4-2.5) |  |  |  |  |
| Fifth quintile | 84.0 | 69.0 (0.82, 0.64-1.04) | 1.1 (0.4-3.2) |  |  |  |  |

aOR, adjusted odds ratio, OR; eq. P, ‘interaction’ P-value testing equality of RR across groups; RCC, remote telephone or video clinical consultation; RR, relative risk. % and RR values calculated using multiple imputation for ethnicity and IMD values, with other missing data excluded. *With adjustment for all variables listed in table; The ‘overall’ aOR value (note not aRR) represents a comparison to the baseline year for a 25-34 year-old woman of white British/Irish ethnicity living in an area with IMD in the first quintile, for other demographic variables such as age the aOR values represent how much greater is the change from baseline year in each group of the variable relative to the respective reference group.

**Table S8:** Time in days from appointment date or sample return date (OPSS testing in CSA-A and CSA-B) for patients with successful chlamydia treatment comparing the ‘mainly face-to face’ (2019) period with the recent ‘mixed consultation model’ (2022) period. Time-to-treatment for chlamydia was evaluated for all cases in CSA-A and CSA-C, and for clinic cases and those OPSS cases obtaining postal treatment in CSA-B.

|  | CSA-A | | CSA-B | | CSA-C | |
| --- | --- | --- | --- | --- | --- | --- |
|  | **2019;** mean±SD | **2022;**  mean±SD (δ, 95%CI) | **2019;** mean±SD | **2022;**  mean±SD (δ, 95%CI) | **2019;** mean±SD | **2022;**  mean±SD (δ, 95%CI) |
| Treated cases by group n/N (%) |  |  |  |  |  |  |
| F2F initial | 2978/4207 (70.8) | 1579/2923 (54.0) | 3397/3946 (86.1) | 1058/3401 (31.1) | 519/564 (92.0) | 65/535 (12.1) |
| OPSS | 1208/4207 (28.7) | 961/2923 (32.9) | 549/3946 (13.9) | 2211/3401 (65.0) | 45/564 (8.0) | 396/535 (74.0) |
| RCC | 21/4207 (0.5) | 383/2923 (13.1) |  | 132/3401 (3.9) |  | 74/535 (13.8) |
| Overall outcome | 7.7±9.3 | 11.3±8.7 (3.59, 3.17-4.01) | 4.4±5.6 | 3.1±3.4 (-1.25, -1.45--1.04) | 5.4±8.0 | 1.0±3.9 |
| Sex grouping *[missing, int. P]* | 0.0% | 0.2%, <0.001 | 0.1% | 0.1%, <0.001 | 0.0% | 0.0% |
| Women | 9.0±9.4 | 11.5±8.2 (2.49, 1.93-3.05) | 4.4±5.0 | 3.1±3.1 (-1.28, -1.57--0.98) | 5.8±8.3 | 0.7±2.7 |
| MSEW | 5.6±8.6 | 10.4±9.3 (4.84, 4.11-5.57) | 3.2±5.3 | 2.6±3.3 (-0.61, -0.99--0.24) | 4.8±7.5 | 1.0±3.9 |
| MSM | 8.6±9.4 | 12.8±9.1 (4.17, 2.96-5.38) | 5.5±6.3 | 3.6±3.7 (-1.89, -2.31--1.47) | 5.3±8.8 | 2.7±8.1 |
| Age *[missing, int. P]* | 0.0% | 0.0%, 0.036 | 0.0% | 0.0%, 0.781 | 0.0% | 0.0% |
| 16-24 | 8.2±9.5 | 11.4±8.6 (3.20, 2.65-3.75) | 4.2±5.5 | 3.0±3.0 (-1.16, -1.49--0.83) | 5.5±8.1 | 0.9±3.3 |
| 25-34 | 6.8±8.6 | 11.2±8.9 (4.44, 3.67-5.22) | 4.2±5.6 | 2.9±3.1 (-1.27, -1.60--0.95) | 4.7±7.1 | 0.9±5.2 |
| 35-49 | 7.6±9.6 | 11.1±8.9 (3.42, 2.06-4.78) | 4.8±5.5 | 3.7±4.4 (-1.07, -1.57--0.56) | 6.4±10.1 | 1.2±3.3 |
| ≥50 | 8.0±8.9 | 12.6±7.8 (4.64, 1.94-7.34) | 5.7±6.2 | 3.6±3.1 (-2.08, -3.05--1.12) | 5.6±8.5 | 5.6±7.5 |
| Ethnicity *[missing, int. P]* | 6.8% | 16.4%, 0.190 | 30.8% | 8.9%, 0.306 |  |  |
| White - Br/Ir | 7.9±9.1 | 11.8±8.6 (3.95, 3.26-4.64) | 4.1±5.2 | 3.1±3.1 (-0.98, -1.34--0.63) |  |  |
| White - other | 8.4±9.8 | 12.3±9.1 (3.90, 1.68-6.12) | 5.0±6.0 | 3.3±3.5 (-1.70, -2.21--1.20) |  |  |
| Black African | 7.9±9.3 | 10.2±8.5 (2.25, 0.98-3.51) | 4.4±6.0 | 3.0±3.6 (-1.41, -2.21--0.60) |  |  |
| Black Carib/oth | 7.1±9.0 | 11.4±8.9 (4.33, 3.36-5.31) | 4.2±6.0 | 2.8±3.1 (-1.32, -2.08--0.56) |  |  |
| Asian | 8.1±9.3 | 11.2±8.6 (3.08, 1.72-4.45) | 4.7±5.5 | 3.2±3.3 (-1.45, -2.27--0.62) |  |  |
| Any other | 9.9±10.7 | 12.8±10.8 (2.84, -1.62-7.30) | 4.0±5.3 | 3.2±3.1 (-0.81, -1.89-0.26) |  |  |
| Mixed | 7.5±9.7 | 10.6±8.3 (3.04, 1.76-4.33) | 4.1±5.1 | 3.2±3.9 (-0.87, -1.60--0.14) |  |  |
| IMD *[missing, int. P]* | 6.4% | 9.1%, 0.328 | 0.9% | 0.2%, 0.533 |  |  |
| First quintile [most deprived] | 7.7±9.4 | 11.1±8.7 (3.40, 2.83-3.97) | 4.3±5.6 | 3.2±3.5 (-1.17, -1.64--0.70) |  |  |
| Second quintile | 7.5±8.8 | 11.7±9.1 (4.24, 3.28-5.19) | 4.6±5.8 | 3.1±3.2 (-1.49, -1.83--1.14) |  |  |
| Third quintile | 9.0±9.9 | 12.1±8.6 (3.12, 1.88-4.37) | 4.2±5.4 | 3.1±3.5 (-1.07, -1.50--0.65) |  |  |
| Fourth quintile | 7.2±8.5 | 9.9±7.1 (2.76, 1.07-4.45) | 4.3±5.0 | 3.2±3.2 (-1.08, -1.63--0.54) |  |  |
| Fifth quintile | 6.6±8.2 | 11.3±8.5 (4.69, 2.61-6.76) | 4.3±5.5 | 3.3±3.8 (-1.04, -1.97--0.12) |  |  |

Direct comparison of 2019 vs 2022 omitted for CSA-C, due to lack of knowledge of sample return date for OPSS testers. RCC, remote telephone or video clinical consultation.

**Table S9:** Time in days from appointment date or sample return date (OPSS testing in CSA-A) for patients with successful gonorrhoea treatment comparing the ‘mainly face-to face’ (2019) period with the recent ‘mixed consultation model’ (2022) period. Time-to-treatment for gonorrhoea was evaluated for all cases in CSA-A and CSA-C, and for clinic cases only in CSA-B.

|  | CSA-A | | CSA-B | | CSA-C | |
| --- | --- | --- | --- | --- | --- | --- |
|  | **2019;** mean±SD | **2022;**  mean±SD (δ, 95%CI) | **2019;** mean±SD | **2022;**  mean±SD (δ, 95%CI) | **2019;** mean±SD | **2022;**  mean±SD (δ, 95%CI) |
| Treated cases by group n/N (%) |  |  |  |  |  |  |
| F2F initial | 1544/1988 (77.7) | 1275/1986 (64.2) | 1276/1276 (100.0) | 665/709 (93.8) | 69/80 (86.2) | 24/118 (20.3) |
| OPSS | 424/1988 (21.3) | 417/1986 (21.0) |  |  | 11/80 (13.8) | 70/118 (59.3) |
| RCC | 20/1988 (1.0) | 294/1986 (14.8) |  | 44/709 (6.2) |  | 24/118 (20.3) |
| Overall outcome | 5.9±7.9 | 8.4±9.5 (2.46, 1.91-3.01) | 3.3±5.6 | 1.7±4.4 (-1.61, -2.06--1.17) | 4.6±6.4 | 2.8±4.9 |
| Sex grouping *[missing, int. P]* | 0.4% | 0.6%, <0.001 | 0.1% | 0.1%, 0.001 | 0.0% | 0.0% |
| Women | 7.2±8.0 | 10.7±8.9 (3.52, 2.63-4.42) | 3.4±4.3 | 2.3±3.8 (-1.15, -1.96--0.35) | 6.2±7.2 | 2.5±4.1 |
| MSEW | 4.6±7.9 | 4.1±7.5 (-0.52, -1.37-0.33) | 1.7±4.1 | 1.3±4.2 (-0.39, -1.13-0.35) | 2.6±4.4 | 3.2±5.6 |
| MSM | 5.8±7.5 | 10.6±10.3 (4.81, 3.82-5.80) | 3.9±6.4 | 1.6±4.6 (-2.27, -2.96--1.58) | 3.6±6.0 | 2.8±5.4 |
| Age *[missing, int. P]* | 0.0% | 0.0%, 0.286 | 0.0% | 0.0%, 0.344 | 0.0% | 0.0% |
| 16-24 | 6.7±8.1 | 8.6±9.5 (1.90, 1.06-2.75) | 2.7±4.7 | 1.7±4.3 (-1.04, -1.86--0.22) | 6.7±7.0 | 2.9±4.9 |
| 25-34 | 5.5±7.7 | 8.2±9.4 (2.71, 1.79-3.64) | 3.4±5.7 | 1.6±4.2 (-1.85, -2.58--1.12) | 1.4±4.3 | 2.0±4.5 |
| 35-49 | 5.1±7.7 | 8.0±9.4 (2.97, 1.68-4.26) | 3.4±5.9 | 2.0±4.9 (-1.35, -2.28--0.42) | 4.2±6.0 | 2.6±4.5 |
| ≥50 | 4.7±7.1 | 8.9±10.0 (4.12, 1.73-6.51) | 4.0±6.5 | 1.0±3.1 (-3.07, -4.49--1.64) | 1.0±2.2 | 3.9±6.0 |
| Ethnicity *[missing, int. P]* | 7.7% | 18.3%, 0.228 | 36.1% | 26.9%, 0.232 |  |  |
| White - Br/Ir | 6.0±7.5 | 9.3±9.4 (3.27, 2.40-4.13) | 2.9±5.6 | 1.6±4.8 (-1.34, -2.27--0.42) |  |  |
| White - other | 6.3±8.3 | 8.7±9.3 (2.45, -0.29-5.19) | 4.0±6.0 | 1.4±3.7 (-2.65, -3.63--1.66) |  |  |
| Black African | 5.5±7.5 | 6.7±9.2 (1.19, -0.90-3.29) | 3.0±4.9 | 2.5±5.3 (-0.44, -2.41-1.52) |  |  |
| Black Carib/oth | 6.0±8.1 | 7.3±9.3 (1.34, 0.07-2.62) | 3.1±5.3 | 1.4±4.1 (-1.64, -3.12--0.16) |  |  |
| Asian | 5.5±8.0 | 7.5±9.4 (2.07, 0.39-3.75) | 3.7±5.8 | 1.7±3.7 (-2.04, -4.05--0.02) |  |  |
| Any other | 7.0±7.5 | 11.2±10.2 (4.17, -0.88-9.23) | 2.6±4.6 | 3.7±5.4 (1.10, -2.10-4.30) |  |  |
| Mixed | 5.9±8.6 | 8.7±9.7 (2.80, 1.12-4.49) | 2.9±5.3 | 1.7±3.8 (-1.22, -2.79-0.35) |  |  |
| IMD *[missing, int. P]* | 5.6% | 7.9%, 0.546 | 1.2% | 1.1%, 0.266 |  |  |
| First quintile [most deprived] | 5.7±7.8 | 7.9±9.5 (2.18, 1.47-2.89) | 3.9±7.0 | 1.6±4.2 (-2.30, -3.37--1.23) |  |  |
| Second quintile | 6.4±8.3 | 9.4±9.3 (2.97, 1.67-4.27) | 3.0±4.6 | 1.8±4.8 (-1.14, -1.85--0.43) |  |  |
| Third quintile | 6.9±8.1 | 9.5±9.3 (2.55, 0.97-4.13) | 3.2±5.3 | 1.4±3.8 (-1.73, -2.57--0.89) |  |  |
| Fourth quintile | 4.5±6.7 | 8.4±9.2 (3.98, 1.67-6.28) | 3.4±6.0 | 1.3±3.2 (-2.18, -3.36--1.00) |  |  |
| Fifth quintile | 6.6±7.3 | 8.4±9.4 (1.83, -0.91-4.57) | 3.1±6.4 | 2.8±5.7 (-0.30, -2.92-2.33) |  |  |

Direct comparison of 2019 vs 2022 omitted for CSA-C, due to lack of knowledge of sample return date for OPSS testers. RCC, remote telephone or video clinical consultation.

**Table S10:** Contraceptive service access rates comparing the ‘mainly face-to face’ (2019) period with the recent ‘mixed consultation model’ (2022) period

|  | CSA-A | | | CSA-B | | | CSA-C | | |
| --- | --- | --- | --- | --- | --- | --- | --- | --- | --- |
|  | **2019;** IR/100PY | **2022;** IR/100PY (IRR, 95%CI) | **2022**; aIRR (95%CI) | **2019;** IR/100PY | **2022;** IR/100PY (IRR, 95%CI) | **2022**; aIRR (95%CI) | **2019;** IR/100PY | **2022;** IR/100PY (IRR, 95%CI) | **2022**; aIRR (95%CI) |
| Overall access rate | 4.5 | 3.3 (0.7, 0.7-0.7) | 0.8 (0.7-0.8) | 9.5 | 6.2 (0.7, 0.6-0.7) | 0.7 (0.6-0.7) | 13 | 9.8 (0.8, 0.8-0.8) | 0.9 (0.8-1.0) |
| Rate by group |  |  |  |  |  |  |  |  |  |
| F2F initial | 4.4 | 1.9 |  | 9.5 | 4.3 |  | 12 | 3.1 |  |
| RCC | .16 | 1.4 |  | .02 | 1.9 |  | .53 | 6.7 |  |
| Age *[missing, int. P]* | 0.0% | 0.0%, <0.001 |  | 0.0% | 0.0%, <0.001 |  | 0.0% | 0.0%, <0.001 |  |
| 16-24 | 8.4 | 4.9 (0.6, 0.6-0.6) | 0.7 (0.7-0.7) | 14 | 7.8 (0.5, 0.5-0.6) | 0.8 (0.8-0.9) | 26 | 16 (0.6, 0.6-0.7) | 0.7 (0.6-0.7) |
| 25-34 | 4.6 | 3.8 (0.8, 0.8-0.9) | Ref. | 13 | 8.1 (0.6, 0.6-0.7) | Ref. | 12 | 11 (0.9, 0.9-1.0) | Ref. |
| 35-49 | 1.8 | 1.9 (1.0, 1.0-1.1) | 1.2 (1.1-1.3) | 4.2 | 3.7 (0.9, 0.9-0.9) | 1.4 (1.3-1.4) | 5.4 | 5.4 (1.0, 0.9-1.1) | 1.1 (1.0-1.2) |
| Ethnicity *[missing, int. P]* | 13.8% | 32.8%, <0.001 |  | 33.7% | 30.0%, <0.001 |  | 2.3% | 1.8%, 0.002 |  |
| White - Br/Ir | 4.9 | 3.2 (0.7, 0.6-0.7) | Ref. | 13 | 7.5 (0.6, 0.6-0.6) | Ref. | 13 | 10 (0.8, 0.8-0.8) | Ref. |
| White - other | 5.2 | 3.1 (0.6, 0.5-0.7) | 0.9 (0.8-1.0) | 8.4 | 5.4 (0.6, 0.6-0.7) | 1.1 (1.0-1.1) | 12 | 6.8 (0.6, 0.5-0.7) | 0.7 (0.6-0.9) |
| Black African | 6.1 | 5.5 (0.9, 0.8-1.0) | 1.4 (1.3-1.5) | 9.5 | 6.9 (0.7, 0.7-0.8) | 1.2 (1.1-1.3) |  |  |  |
| Black Carib/oth | 13 | 12 (0.9, 0.8-1.0) | 1.4 (1.3-1.5) | 16 | 11 (0.7, 0.6-0.8) | 1.1 (1.0-1.3) |  |  |  |
| Asian | 2.1 | 1.6 (0.7, 0.7-0.8) | 1.1 (1.0-1.2) | 5 | 3.9 (0.8, 0.7-0.8) | 1.3 (1.2-1.4) |  |  |  |
| Any other | 1.9 | 1.4 (0.7, 0.6-0.9) | 1.1 (0.9-1.3) | 2.6 | 2.2 (0.9, 0.8-1.0) | 1.4 (1.2-1.6) | 5.6 | 3.9 (0.7, 0.6-0.9) | 0.9 (0.7-1.1) |
| Mixed | 10 | 7.8 (0.8, 0.7-0.8) | 1.2 (1.1-1.3) | 16 | 11 (0.7, 0.6-0.7) | 1.2 (1.1-1.3) |  |  |  |
| IMD *[missing, int. P]* | 10.0% | 11.4%, <0.001 |  | 0.7% | 0.7%, <0.001 |  | 8.8% | 6.1%, 0.304 |  |
| First quintile | 4.7 | 3.6 (0.8, 0.7-0.8) | Ref. | 8.3 | 6 (0.7, 0.7-0.8) | Ref. | 17 | 13 (0.7, 0.7-0.8) | Ref. |
| Second quintile | 4.4 | 3.2 (0.7, 0.7-0.8) | 1.0 (0.9-1.1) | 9 | 5.9 (0.7, 0.6-0.7) | 0.9 (0.9-1.0) | 12 | 9.1 (0.8, 0.7-0.8) | 1.0 (0.9-1.1) |
| Third quintile | 3.9 | 2.8 (0.7, 0.7-0.8) | 1.0 (0.9-1.0) | 8.7 | 5.6 (0.6, 0.6-0.7) | 0.9 (0.8-0.9) | 12 | 9.5 (0.8, 0.8-0.9) | 1.1 (1.0-1.2) |
| Fourth quintile | 3.6 | 2.5 (0.7, 0.6-0.8) | 0.9 (0.8-1.1) | 8.1 | 5 (0.6, 0.6-0.7) | 0.9 (0.8-0.9) | 11 | 9 (0.8, 0.7-0.8) | 1.1 (1.0-1.2) |
| Fifth quintile | 4.6 | 2.5 (0.5, 0.5-0.6) | 0.7 (0.6-0.8) | 8.2 | 5.1 (0.6, 0.6-0.7) | 0.8 (0.8-0.9) | 9.8 | 6.9 (0.7, 0.6-0.8) | 0.9 (0.8-1.1) |

IR and IRR values calculated using multiple imputation for missing covariables. RCC, remote telephone or video clinical consultation.

**Table S11:** Proportion of women aged 16–49 attending for contraceptive care with uptake of long-acting contraception, comparing the ‘mainly face-to face’ (2019) period with ‘mixed consultation model’ (2022) period. Change is estimated within categories (RR; 2022 *vs* 2019) along with relative change compared to reference category (aOR). This is the same as Table 5 in the main paper, with the addition of multivariable models.

|  | CSA-A | | | CSA-B | | | CSA-C | | |
| --- | --- | --- | --- | --- | --- | --- | --- | --- | --- |
|  | **2019;** n/N (%) | **2022;** n/N (%) (RR, 95%CI) | **2022**; aOR* (95%CI) | **2019;** n/N (%) | **2022;** n/N (%) (RR, 95%CI) | **2022**; aOR* (95%CI) | **2019;** n/N (%) | **2022;** n/N (%) (RR, 95%CI) | **2022**; aOR* (95%CI) |
| Overall outcome | 5978/14883 (40.2) | 2509/10851 (23.1) (0.58, 0.55-0.60) | 0.4 (0.4-0.5) | 8460/28478 (29.7) | 6529/18671 (35.0) (1.18, 1.15-1.21) | 1.2 (1.0-1.3) | 3092/8916 (34.7) | 2548/6942 (36.7) (1.06, 1.01-1.11) | 0.9 (0.7-1.1) |
| Outcome by group |  |  |  |  |  |  |  |  |  |
| F2F initial | 5611/14363 (39.1) | 1169/6255 (18.7) |  | 8457/28427 (29.7) | 5762/12871 (44.8) |  | 2878/8545 (33.7) | 1264/2184 (57.9) |  |
| RCC | 367/520 (70.6) | 1340/4596 (29.2) |  | 3/51 (5.9) | 767/5800 (13.2) |  | 214/371 (57.8) | 1284/4758 (27.0) |  |
| Age *[missing, eq. P]* | 0.0% | 0.0%, <0.001 |  | 0.0% | 0.0%, <0.001 |  | 0.0% | 0.0%, 0.290 |  |
| 16-24 | 2541/7774 (32.7) | 878/4512 (19.5) (0.60, 0.55-0.64) | 1.2 (1.0-1.4) | 2456/9552 (25.7) | 1613/5166 (31.2) (1.21, 1.15-1.28) | 1.0 (0.9-1.1) | 1495/4744 (31.5) | 983/2965 (33.2) (1.05, 0.98-1.13) | 1.0 (0.8-1.1) |
| 25-34 | 2051/4615 (44.4) | 955/3817 (25.0) (0.56, 0.53-0.60) | Ref. | 3941/13674 (28.8) | 3019/8831 (34.2) (1.19, 1.14-1.23) | Ref. | 864/2468 (35.0) | 844/2269 (37.2) (1.06, 0.98-1.15) | Ref. |
| 35-49 | 1386/2494 (55.6) | 676/2522 (26.8) (0.48, 0.45-0.52) | 0.7 (0.6-0.8) | 2063/5252 (39.3) | 1897/4674 (40.6) (1.03, 0.98-1.08) | 0.8 (0.7-0.9) | 733/1704 (43.0) | 721/1708 (42.2) (0.98, 0.91-1.06) | 0.9 (0.6-1.0) |
| Ethnicity *[missing, eq. P]* | 13.8% | 32.8%, 0.026 |  | 33.7% | 30.0%, 0.613 |  | 2.3% | 1.8%, 0.779 |  |
| White - Br/Ir | 40.8 | 25.5 (0.62, 0.59-0.67) | Ref. | 32.0 | 36.8 (1.15, 1.10-1.20) | Ref. | 34.7 | 36.8 (1.06, 1.01-1.11) | Ref. |
| White - other | 39.3 | 24.1 (0.61, 0.51-0.73) | 1.1 (0.9-1.5) | 28.8 | 35.7 (1.24, 1.16-1.32) | 1.1 (1.0-1.3) | 32.6 | 31.8 (0.98, 0.76-1.25) | 0.9 (0.6-1.2) |
| Black African | 41.0 | 22.5 (0.55, 0.47-0.64) | 1.0 (0.8-1.3) | 27.3 | 31.0 (1.14, 1.01-1.27) | 1.0 (0.8-1.2) | - | - |  |
| Black Carib/oth | 36.5 | 17.1 (0.47, 0.40-0.55) | 0.9 (0.7-1.1) | 24.2 | 28.9 (1.19, 1.03-1.38) | 1.0 (0.8-1.3) | - | - |  |
| Asian | 41.7 | 22.8 (0.55, 0.49-0.62) | 1.0 (0.8-1.2) | 29.3 | 35.5 (1.21, 1.10-1.33) | 1.1 (0.9-1.3) | - | - |  |
| Any other | 43.9 | 26.4 (0.60, 0.45-0.80) | 1.1 (0.7-1.7) | 23.6 | 29.8 (1.26, 0.99-1.61) | 1.1 (0.8-1.6) | 36.6 | 40.6 (1.11, 0.81-1.52) | 1.0 (0.6-1.8) |
| Mixed | 38.4 | 22.3 (0.58, 0.50-0.67) | 1.0 (0.8-1.2) | 29.3 | 34.8 (1.19, 1.09-1.30) | 1.0 (0.9-1.2) | - | - |  |
| IMD *[missing, eq. P]* | 10.0% | 11.4%, 0.019 |  | 0.7% | 0.7%, 0.785 |  | 8.8% | 6.1%, 0.057 |  |
| First quintile [most deprived] | 43.3 | 24.1 (0.56, 0.53-0.59) | Ref. | 29.5 | 33.5 (1.14, 1.07-1.22) | Ref. | 36.4 | 34.0 (0.93, 0.83-1.05) | Ref. |
| Second quintile | 38.2 | 21.8 (0.57, 0.52-0.63) | 1.0 (0.9-1.2) | 28.8 | 34.2 (1.19, 1.13-1.24) | 1.1 (0.9-1.2) | 37.7 | 37.6 (1.00, 0.91-1.09) | 1.1 (0.9-1.4) |
| Third quintile | 38.3 | 21.2 (0.55, 0.49-0.63) | 1.0 (0.8-1.2) | 29.9 | 36.0 (1.20, 1.14-1.27) | 1.1 (1.0-1.2) | 34.0 | 37.8 (1.11, 1.03-1.20) | 1.3 (1.1-1.6) |
| Fourth quintile | 33.9 | 22.8 (0.67, 0.55-0.83) | 1.3 (1.0-1.8) | 30.9 | 36.1 (1.17, 1.09-1.25) | 1.0 (0.9-1.2) | 32.9 | 36.0 (1.09, 1.01-1.19) | 1.3 (1.0-1.6) |
| Fifth quintile | 28.6 | 22.1 (0.77, 0.63-0.95) | 1.6 (1.2-2.1) | 31.2 | 36.6 (1.18, 1.07-1.29) | 1.0 (0.9-1.2) | 31.1 | 37.4 (1.20, 0.98-1.48) | 1.5 (1.0-2.2) |

aOR, adjusted odds ratio; eq. P, ‘interaction’ P-value testing equality of RR for change over time across groups; IMD, index of multiple deprivation; MSEW, men who have sex exclusively with women; MSM, men who have sex with men; RCC, remote telephone or video clinical consultation; RR, relative risk. % and RR values calculated using multiple imputation for ethnicity and IMD values, with other missing data excluded. *With adjustment for all variables listed in table; The ‘overall’ aOR value (note not aRR) represents a comparison to the baseline year for a 25-34 year-old woman of white British/Irish ethnicity living in an area with IMD in the first quintile, for other demographic variables such as age the aOR values represent how much greater is the change from baseline year in each group of the variable relative to the respective reference group.

**Table S12:** Proportion of women aged 16–49 attending for contraceptive care with uptake of long-acting contraception, comparing initial contact via remote care (RC) or face-to-face (F2F) within 2022.

|  | CSA-A | | | CSA-B | | | CSA-C | |
| --- | --- | --- | --- | --- | --- | --- | --- | --- |
|  | F2F; n/N (%) | RC; n/N (%) (RR, 95%CI) | RC; aOR (95%CI) | F2F; n/N (%) | RC; n/N (%) (RR, 95%CI) | RC; aOR (95%CI) | F2F; n/N (%) | RC; n/N (%) (RR, 95%CI) |
| Overall outcome | 1169/6255 (18.7) | 1340/4596 (29.2) (1.56, 1.45-1.67) | 1.8 (1.4-2.2) | 5762/12871 (44.8) | 767/5800 (13.2) (0.30, 0.28-0.32) | 0.2 (0.1-0.2) | 1264/2184 (57.9) | 1284/4758 (27.0) (0.47, 0.44-0.50) |
| Age *[missing, eq. P]* | 0.0% | 0.0%, 0.900 |  | 0.0% | 0.0%, <0.001 |  | 0.0% | 0.0%, <0.001 |
| 16-24 | 506/3022 (16.7) | 372/1490 (25.0) (1.49, 1.32-1.68) | 0.9 (0.7-1.2) | 1461/3502 (41.7) | 152/1664 (9.1) (0.22, 0.19-0.26) | 0.8 (0.7-1.0) | 565/927 (60.9) | 418/2038 (20.5) (0.34, 0.30-0.37) |
| 25-34 | 373/1899 (19.6) | 582/1918 (30.3) (1.54, 1.37-1.74) | Ref. | 2670/5986 (44.6) | 349/2845 (12.3) (0.28, 0.25-0.31) | Ref. | 402/711 (56.5) | 442/1558 (28.4) (0.50, 0.45-0.56) |
| 35-49 | 290/1334 (21.7) | 386/1188 (32.5) (1.49, 1.31-1.71) | 1.0 (0.8-1.2) | 1631/3383 (48.2) | 266/1291 (20.6) (0.43, 0.38-0.48) | 1.6 (1.3-2.0) | 297/546 (54.4) | 424/1162 (36.5) (0.67, 0.60-0.75) |
| Ethnicity *[missing, eq. P]* | 24.6% | 44.0%, 0.561 |  | 33.1% | 23.2%, 0.352 |  |  |  |
| White - Br/Ir | 19.7 | 31.6 (1.60, 1.43-1.80) | Ref. | 47.9 | 13.5 (0.28, 0.25-0.32) | Ref. |  |  |
| White - other | 22.2 | 27.0 (1.22, 0.87-1.71) | 0.7 (0.4-1.2) | 45.5 | 13.8 (0.30, 0.26-0.35) | 1.1 (0.8-1.3) |  |  |
| Black African | 19.5 | 29.2 (1.50, 1.16-1.93) | 0.9 (0.6-1.4) | 37.3 | 14.1 (0.38, 0.29-0.49) | 1.5 (1.0-2.3) |  |  |
| Black Carib/oth | 14.8 | 21.1 (1.42, 1.11-1.82) | 0.8 (0.6-1.2) | 36.8 | 11.6 (0.31, 0.23-0.44) | 1.2 (0.8-1.9) |  |  |
| Asian | 17.7 | 30.5 (1.73, 1.41-2.12) | 1.1 (0.8-1.6) | 46.2 | 11.4 (0.25, 0.18-0.33) | 0.9 (0.6-1.3) |  |  |
| Any other | 19.7 | 35.2 (1.79, 0.96-3.36) | 1.3 (0.5-3.0) | 38.9 | 14.5 (0.37, 0.25-0.55) | 1.5 (0.9-2.6) |  |  |
| Mixed | 19.5 | 26.4 (1.35, 1.06-1.72) | 0.8 (0.6-1.2) | 43.8 | 12.7 (0.29, 0.23-0.37) | 1.1 (0.8-1.5) |  |  |
| IMD *[missing, eq. P]* | 12.8% | 9.4%, 0.763 |  | 0.7% | 0.8%, 0.690 |  |  |  |
| First quintile [most deprived] | 19.9 | 29.8 (1.50, 1.37-1.64) | Ref. | 43.2 | 12.4 (0.29, 0.24-0.34) | Ref. |  |  |
| Second quintile | 16.9 | 28.4 (1.68, 1.41-2.00) | 1.1 (0.9-1.4) | 43.0 | 13.6 (0.32, 0.28-0.36) | 1.2 (0.9-1.5) |  |  |
| Third quintile | 16.5 | 27.6 (1.68, 1.35-2.09) | 1.1 (0.8-1.6) | 46.1 | 13.3 (0.29, 0.25-0.33) | 1.0 (0.8-1.3) |  |  |
| Fourth quintile | 18.8 | 28.5 (1.52, 1.10-2.09) | 1.0 (0.7-1.6) | 47.1 | 13.5 (0.29, 0.24-0.34) | 1.0 (0.8-1.4) |  |  |
| Fifth quintile | 17.6 | 29.1 (1.65, 1.17-2.33) | 1.1 (0.7-1.8) | 48.6 | 13.0 (0.27, 0.21-0.34) | 0.9 (0.6-1.3) |  |  |

aOR, adjusted OR; eq. P, P-value testing equality of RR across groups; RR, relative risk. % and RR values calculated using multiple imputation for ethnicity and IMD values, with other missing data excluded. *With adjustment for all variables listed in table; The ‘overall’ aOR value (note not aRR) represents a comparison to the baseline year for a 25-34 year-old woman of white British/Irish ethnicity living in an area with IMD in the first quintile, for other demographic variables such as age the aOR values represent how much greater is the change from baseline year in each group of the variable relative to the respective reference group.

**Table S13:** Proportion of all episodes of care in which it was recorded that recent sexual assault was disclosed, comparing the ‘mainly face-to face’ (2019) period with ‘mixed consultation model’ (2022) period. Change is estimated within categories (RR; 2022 *vs* 2019) along with relative change compared to reference category (aOR).

|  | CSA-A | | | CSA-B | | | CSA-C | |
| --- | --- | --- | --- | --- | --- | --- | --- | --- |
|  | **2019;** n/N (%) | **2022;** n/N (%) (RR, 95%CI) | **2022**; aOR* (95%CI) | **2019;** n/N (%) | **2022;** n/N (%) (RR, 95%CI) | **2022**; aOR* (95%CI) | **2019;** n/N (%) | **2022;** n/N (%) (RR, 95%CI) |
| Overall outcome | 1791/91844 (2.0) | 1474/61789 (2.4) (1.22, 1.14-1.31) | 0.9 (0.8-1.1) | 295/127358 (0.2) | 509/117406 (0.4) (1.87, 1.62-2.16) | 1.6 (1.0-2.6) | 6/17685 (0.0) | 25/17714 (0.1) (4.16, 1.71-10.14) |
| Outcome by group |  |  |  |  |  |  |  |  |
| F2F initial | 1637/57179 (2.9) | 1061/23873 (4.4) |  | 187/99332 (0.2) | 135/47516 (0.3) |  | 6/16637 (0.0) | 7/4327 (0.2) |
| OPSS | 97/32766 (0.3) | 126/26336 (0.5) |  | 108/27911 (0.4) | 363/59351 (0.6) |  | 0/679 (0.0) | 14/5750 (0.2) |
| RCC | 57/1899 (3.0) | 287/11580 (2.5) |  | 0/115 (0.0) | 11/10539 (0.1) |  | 0/369 (0.0) | 4/7637 (0.1) |
| Sex grouping *[missing, eq. P]* | 0.2% | 0.4%, 0.049 |  | 0.1% | 0.1%, <0.001 |  |  |  |
| Women | 1417/58249 (2.4) | 1150/36926 (3.1) (1.28, 1.18-1.38) | Ref. | 201/71830 (0.3) | 415/61564 (0.7) (2.41, 2.03-2.86) | Ref. |  |  |
| MSEW | 155/24716 (0.6) | 101/17606 (0.6) (0.91, 0.71-1.18) | 0.7 (0.5-0.9) | 51/29332 (0.2) | 36/30015 (0.1) (0.69, 0.45-1.06) | 0.3 (0.2-0.5) |  |  |
| MSM | 219/8879 (2.5) | 223/7257 (3.1) (1.25, 1.03-1.51) | 1.1 (0.9-1.4) | 43/26196 (0.2) | 58/25827 (0.2) (1.37, 0.92-2.03) | 0.6 (0.4-1.0) |  |  |
| Age *[missing, eq. P]* | 0.0% | 0.0%, <0.001 |  | 0.0% | 0.0%, 0.021 |  |  |  |
| 16-24 | 875/40212 (2.2) | 737/22947 (3.2) (1.48, 1.34-1.63) | 1.5 (1.2-1.7) | 120/30535 (0.4) | 248/25336 (1.0) (2.49, 2.00-3.10) | 1.4 (1.0-2.0) |  |  |
| 25-34 | 568/31238 (1.8) | 431/22679 (1.9) (1.05, 0.92-1.19) | Ref. | 120/56577 (0.2) | 173/51118 (0.3) (1.60, 1.26-2.02) | Ref. |  |  |
| 35-49 | 286/16321 (1.8) | 255/12902 (2.0) (1.13, 0.95-1.34) | 1.0 (0.8-1.3) | 44/29250 (0.2) | 74/28665 (0.3) (1.72, 1.17-2.52) | 1.1 (0.7-1.8) |  |  |
| ≥50 | 62/4073 (1.5) | 51/3261 (1.6) (1.03, 0.70-1.51) | 1.0 (0.6-1.5) | 11/10996 (0.1) | 14/12287 (0.1) (1.14, 0.52-2.51) | 0.9 (0.4-2.1) |  |  |
| Ethnicity *[missing, eq. P]* | 11.1% | 20.5%, 0.158 |  | 26.2% | 12.7%, 0.667 |  |  |  |
| White - Br/Ir | 2.2 | 2.5 (1.11, 1.01-1.23) | Ref. | 0.3 | 0.4 (1.70, 1.35-2.13) | Ref. |  |  |
| White - other | 1.9 | 2.7 (1.39, 1.02-1.90) | 1.3 (0.9-1.8) | 0.2 | 0.3 (1.94, 1.32-2.87) | 1.2 (0.8-1.9) |  |  |
| Black African | 1.5 | 2.4 (1.57, 1.17-2.11) | 1.4 (1.0-2.0) | 0.2 | 0.5 (2.11, 1.27-3.51) | 1.3 (0.7-2.3) |  |  |
| Black Carib/oth | 1.6 | 2.2 (1.37, 1.10-1.70) | 1.3 (1.0-1.7) | 0.3 | 0.4 (1.35, 0.76-2.39) | 0.8 (0.4-1.5) |  |  |
| Asian | 1.3 | 1.6 (1.31, 1.02-1.68) | 1.3 (0.9-1.7) | 0.2 | 0.5 (2.22, 1.23-4.02) | 1.3 (0.7-2.5) |  |  |
| Any other | 1.6 | 1.4 (0.90, 0.45-1.79) | 0.9 (0.4-1.8) | 0.2 | 0.6 (3.40, 1.31-8.87) | 2.0 (0.7-5.4) |  |  |
| Mixed | 2.4 | 3.2 (1.33, 1.08-1.64) | 1.2 (0.9-1.5) | 0.3 | 0.6 (2.01, 1.27-3.20) | 1.1 (0.7-1.9) |  |  |
| IMD *[missing, eq. P]* | 6.9% | 6.9%, 0.890 |  | 0.8% | 0.5%, 0.437 |  |  |  |
| First quintile [most deprived] | 1.9 | 2.3 (1.21, 1.09-1.34) | Ref. | 0.2 | 0.4 (1.72, 1.23-2.41) | Ref. |  |  |
| Second quintile | 2.2 | 2.6 (1.20, 1.04-1.39) | 1.0 (0.8-1.2) | 0.2 | 0.4 (1.64, 1.29-2.09) | 1.0 (0.6-1.5) |  |  |
| Third quintile | 2.0 | 2.4 (1.21, 1.01-1.45) | 1.0 (0.8-1.3) | 0.2 | 0.4 (2.30, 1.68-3.15) | 1.4 (0.9-2.2) |  |  |
| Fourth quintile | 1.5 | 2.1 (1.43, 1.04-1.98) | 1.2 (0.9-1.8) | 0.3 | 0.5 (1.91, 1.33-2.74) | 1.1 (0.7-1.9) |  |  |
| Fifth quintile | 2.1 | 2.7 (1.27, 0.95-1.70) | 1.1 (0.8-1.6) | 0.2 | 0.5 (2.40, 1.38-4.19) | 1.5 (0.8-2.9) |  |  |

aOR, adjusted odds ratio, OR; eq. P, ‘interaction’ P-value testing equality of RR across groups; RCC, remote telephone or video clinical consultation; RR, relative risk. % and RR values calculated using multiple imputation for ethnicity and IMD values, with other missing data excluded. *With adjustment for all variables listed in table; The ‘overall’ aOR value (note not aRR) represents a comparison to the baseline year for a 25-34 year-old woman of white British/Irish ethnicity living in an area with IMD in the first quintile, for other demographic variables such as age the aOR values represent how much greater is the change from baseline year in each group of the variable relative to the respective reference group.

**Table S14:** Proportion of all episodes of care in which it was recorded that recent sexual assault was disclosed, comparing initial contact via remote care (RC) or face-to-face (F2F) within 2022.

|  | CSA-A | | | CSA-B | | | CSA-C | |
| --- | --- | --- | --- | --- | --- | --- | --- | --- |
|  | F2F; n/N (%) | RC; n/N (%) (RR, 95%CI) | RC; aOR (95%CI) | F2F; n/N (%) | RC; n/N (%) (RR, 95%CI) | RC; aOR (95%CI) | F2F; n/N (%) | RC; n/N (%) (RR, 95%CI) |
| Overall outcome | 1061/23873 (4.4) | 413/37916 (1.1) (0.25, 0.22-0.27) | 0.2 (0.1-0.2) | 135/47516 (0.3) | 374/69890 (0.5) (1.88, 1.55-2.30) | 0.6 (0.3-1.1) | 7/4327 (0.2) | 18/13387 (0.1) (0.83, 0.35-1.99) |
| Outcome by group |  |  |  |  |  |  |  |  |
| OPSS | --- | 126/26336 (0.5) |  | --- | 363/59351 (0.6) |  |  |  |
| RCC | --- | 287/11580 (2.5) |  | --- | 11/10539 (0.1) |  |  |  |
| Sex grouping *[missing, eq. P]* | 0.4% | 0.4%, <0.001 |  | 0.1% | 0.0%, 0.014 |  |  |  |
| Women | 833/12331 (6.8) | 317/24595 (1.3) (0.19, 0.17-0.22) | Ref. | 113/21280 (0.5) | 302/40284 (0.7) (1.41, 1.14-1.75) | Ref. |  |  |
| MSEW | 83/8393 (1.0) | 18/9213 (0.2) (0.20, 0.12-0.33) | 1.0 (0.6-1.7) | 9/13206 (0.1) | 27/16809 (0.2) (2.36, 1.10-5.04) | 1.5 (0.7-3.3) |  |  |
| MSM | 145/3149 (4.6) | 78/4108 (1.9) (0.41, 0.31-0.54) | 2.1 (1.5-2.9) | 13/13030 (0.1) | 45/12797 (0.4) (3.52, 1.90-6.55) | 2.3 (1.1-4.7) |  |  |
| Age *[missing, eq. P]* | 0.0% | 0.0%, <0.001 |  | 0.0% | 0.0%, 0.507 |  |  |  |
| 16-24 | 562/8249 (6.8) | 175/14698 (1.2) (0.17, 0.15-0.21) | 0.8 (0.6-1.0) | 55/6993 (0.8) | 193/18343 (1.1) (1.34, 0.99-1.81) | 1.1 (0.7-1.8) |  |  |
| 25-34 | 295/8125 (3.6) | 136/14554 (0.9) (0.26, 0.21-0.31) | Ref. | 45/16432 (0.3) | 128/34686 (0.4) (1.35, 0.96-1.90) | Ref. |  |  |
| 35-49 | 170/5764 (2.9) | 85/7138 (1.2) (0.40, 0.31-0.52) | 1.5 (1.1-2.1) | 25/14638 (0.2) | 49/14027 (0.3) (2.05, 1.25-3.35) | 1.4 (0.7-2.5) |  |  |
| ≥50 | 34/1735 (2.0) | 17/1526 (1.1) (0.26, 0.21-0.31) | 2.0 (1.1-3.6) | 10/9453 (0.1) | 4/2834 (0.1) (1.35, 0.96-1.90) | 0.7 (0.2-2.6) |  |  |
| Ethnicity *[missing, eq. P]* | 25.6% | 17.3%, 0.141 |  | 25.4% | 4.0%, 0.631 |  |  |  |
| White - Br/Ir | 5.9 | 1.1 (0.19, 0.16-0.22) | Ref. | 0.3 | 0.5 (1.68, 1.21-2.32) | Ref. |  |  |
| White - other | 4.4 | 1.3 (0.29, 0.16-0.50) | 1.4 (0.8-2.7) | 0.2 | 0.4 (2.55, 1.45-4.46) | 1.6 (0.8-3.1) |  |  |
| Black African | 3.5 | 1.0 (0.28, 0.17-0.48) | 1.6 (0.9-2.9) | 0.3 | 0.7 (2.33, 1.16-4.68) | 1.8 (0.8-4.0) |  |  |
| Black Carib/oth | 3.5 | 1.0 (0.28, 0.19-0.40) | 1.4 (0.9-2.1) | 0.3 | 0.5 (1.46, 0.57-3.70) | 1.1 (0.4-3.0) |  |  |
| Asian | 2.6 | 0.6 (0.25, 0.15-0.40) | 1.2 (0.7-2.1) | 0.4 | 0.5 (1.23, 0.62-2.45) | 0.8 (0.4-1.8) |  |  |
| Any other | 2.6 | 0.6 (0.22, 0.06-0.80) | 1.1 (0.3-4.2) | 0.3 | 0.7 (2.04, 0.74-5.65) | 1.2 (0.4-3.7) |  |  |
| Mixed | 5.6 | 1.6 (0.30, 0.22-0.41) | 1.6 (1.1-2.4) | 0.3 | 0.7 (2.53, 1.29-4.96) | 1.9 (0.9-4.2) |  |  |
| IMD *[missing, eq. P]* | 12.1% | 3.7%, 0.010 |  | 1.1% | 0.1%, 0.320 |  |  |  |
| First quintile [most deprived] | 3.8 | 1.2 (0.30, 0.25-0.35) | Ref. | 0.4 | 0.5 (1.29, 0.84-1.97) | Ref. |  |  |
| Second quintile | 5.1 | 1.1 (0.21, 0.16-0.27) | 0.7 (0.5-1.0) | 0.3 | 0.5 (1.95, 1.38-2.75) | 1.6 (0.9-2.8) |  |  |
| Third quintile | 5.6 | 1.0 (0.18, 0.13-0.24) | 0.7 (0.5-1.0) | 0.2 | 0.6 (2.52, 1.62-3.92) | 2.1 (1.1-4.0) |  |  |
| Fourth quintile | 4.7 | 0.9 (0.18, 0.11-0.30) | 0.7 (0.4-1.2) | 0.3 | 0.6 (1.86, 1.12-3.09) | 1.6 (0.8-3.1) |  |  |
| Fifth quintile | 5.3 | 1.1 (0.21, 0.13-0.34) | 0.9 (0.5-1.6) | 0.3 | 0.6 (1.99, 0.95-4.16) | 1.7 (0.7-4.0) |  |  |

aOR, adjusted odds ratio, OR; eq. P, ‘interaction’ P-value testing equality of RR across groups; RCC, remote telephone or video clinical consultation; RR, relative risk. % and RR values calculated using multiple imputation for ethnicity and IMD values, with other missing data excluded. *With adjustment for all variables listed in table; The ‘overall’ aOR value (note not aRR) represents a comparison to the baseline year for a 25-34 year-old woman of white British/Irish ethnicity living in an area with IMD in the first quintile, for other demographic variables such as age the aOR values represent how much greater is the change from baseline year in each group of the variable relative to the respective reference group.
